# Reverse Genetics for Fusogenic Bat-Borne Orthoreovirus Associated with Acute Respiratory Tract Infections in Humans: Role of Outer Capsid Protein σC in Viral Replication and Pathogenesis

**DOI:** 10.1371/journal.ppat.1005455

**Published:** 2016-02-22

**Authors:** Takahiro Kawagishi, Yuta Kanai, Hideki Tani, Masayuki Shimojima, Masayuki Saijo, Yoshiharu Matsuura, Takeshi Kobayashi

**Affiliations:** 1 Laboratory of Viral Replication, International Research Center for Infectious Diseases, Osaka University, Suita, Osaka, Japan; 2 Department of Molecular Virology, Research Institute for Microbial Diseases, Osaka University, Suita, Osaka, Japan; 3 Special Pathogens Laboratory, Department of Virology I, National Institute of Infectious Diseases, Musashimurayama, Tokyo, Japan; University of North Carolina at Chapel Hill, UNITED STATES

## Abstract

Nelson Bay orthoreoviruses (NBVs) are members of the fusogenic orthoreoviruses and possess 10-segmented double-stranded RNA genomes. NBV was first isolated from a fruit bat in Australia more than 40 years ago, but it was not associated with any disease. However, several NBV strains have been recently identified as causative agents for respiratory tract infections in humans. Isolation of these pathogenic bat reoviruses from patients suggests that NBVs have evolved to propagate in humans in the form of zoonosis. To date, no strategy has been developed to rescue infectious viruses from cloned cDNA for any member of the fusogenic orthoreoviruses. In this study, we report the development of a plasmid-based reverse genetics system free of helper viruses and independent of any selection for NBV isolated from humans with acute respiratory infection. cDNAs corresponding to each of the 10 full-length RNA gene segments of NBV were cotransfected into culture cells expressing T7 RNA polymerase, and viable NBV was isolated using a plaque assay. The growth kinetics and cell-to-cell fusion activity of recombinant strains, rescued using the reverse genetics system, were indistinguishable from those of native strains. We used the reverse genetics system to generate viruses deficient in the cell attachment protein σC to define the biological function of this protein in the viral life cycle. Our results with σC-deficient viruses demonstrated that σC is dispensable for cell attachment in several cell lines, including murine fibroblast L929 cells but not in human lung epithelial A549 cells, and plays a critical role in viral pathogenesis. We also used the system to rescue a virus that expresses a yellow fluorescent protein. The reverse genetics system developed in this study can be applied to study the propagation and pathogenesis of pathogenic NBVs and in the generation of recombinant NBVs for future vaccines and therapeutics.

## Author Summary

Nelson Bay orthoreoviruses (NBVs) are members of the fusogenic orthoreoviruses that have various host species, including reptiles, birds, and mammals. Recently, several NBV strains have been isolated from patients with acute respiratory tract infections. Isolation of these pathogenic reoviruses raises concerns about the potential emerging infections of bat-borne orthoreoviruses in humans. The development of an entirely plasmid-based reverse genetics system for double-stranded RNA viruses has trailed other systems of major animal RNA virus groups because of the technical complexities involved in the manipulation of genomes composed of 10 or more segments. In this study, we developed a plasmid-based reverse genetics system for a pathogenic NBV strain. We used this system to generate viruses incapable of expressing the cell attachment protein σC and to rescue a replication-competent virus that expresses a yellow fluorescent protein. Our studies using σC-deficient viruses suggest that NBVs may engage multiple independent viral ligands and cellular receptors for efficient cell attachment and viral pathogenesis, thus providing new insight into the biology of orthoreoviruses. The reverse genetics approach described in this study can be exploited for fusogenic orthoreovirus biology and used to develop vaccines, diagnostics, and therapeutics.

## Introduction

Members of the genus *Orthoreovirus* belonging to the family *Reoviridae* are nonenveloped viruses. Their genomes contain 10-segmented double-stranded RNA (dsRNA) divided into three classes based on their sizes: large (L1–L3), medium (M1–M3), and small (S1–S4). The orthoreoviruses are classified into fusogenic and nonfusogenic subgroups based on their ability to induce cell-to-cell fusion during cell culture [1]. The fusogenic subgroup comprises the avian orthoreovirus (ARV), baboon orthoreovirus (BRV), reptilian orthoreovirus, Broome reovirus (BroV), and Nelson Bay orthoreovirus (NBV), whereas the nonfusogenic subgroup comprises the prototypical mammalian orthoreovirus (MRV) [1–3]. Nonfusogenic MRVs are quite common and generally asymptomatic in humans. Although natural infections involving fusogenic orthoreoviruses cause severe diseases in infected animals, infections involving these orthoreoviruses in humans have not been reported. However, in 2006, the Melaka (Mel) virus, a new fusogenic orthoreovirus, was isolated from a patient with acute respiratory tract infection in Malaysia [4]. This newly isolated virus is genetically related to the NBV strains Nelson Bay (NB) and Pulau, which were isolated from fruit bats in Australia and Malaysia, respectively [5, 6]. Subsequently, other related NBV strains have been isolated from patients with severe respiratory illness in Malaysia and Hong Kong [7–10]. Recently, we reported an imported case of a respiratory tract infection associated with NBV strains in a patient who returned to Japan from Bali, Indonesia, in 2007 and termed the strain Miyazaki-Bali/2007 (MB) [11, 12]. Although there is no evidence for direct human-to-human or bat-to-human transmission of MB virus, possible bat-to-human or human-to-human transmissions have been reported in human infections by the Mel, Kampar, and Sikamat viruses [4, 7, 9, 11]. A more recent epidemiological study in Malaysia detected NBVs in the oropharyngeal swab samples of 34 of 200 patients with acute upper respiratory tract infections [13]. These isolates have given rise to increasing concerns about the zoonotic transmission of bat-borne orthoreoviruses in humans.

For the most part, the gene segments of orthoreoviruses are monocistronic and encode a single unique translation product. However, the S1 segments of ARV and NBV encode tricistronic mRNAs containing three partially overlapping open reading frames (ORFs) that are translated into two nonstructural proteins, p10 and p17, and one structural protein, σC [14, 16]. The p10 protein is a fusion-associated small transmembrane (FAST) protein that has been shown to induce cell-to-cell fusion and the syncytium-inducing properties of fusogenic orthoreoviruses [17]. The p17 protein, which is encoded by the second ORF of the S1 segment, has no sequence similarity to the known proteins. Previous studies revealed that the ARV p17 is a CRM-1-independent nucleocytoplasmic shuttling protein that plays an important role in the nuclear process comprising gene transcription and cell growth regulation [18, 19]. The σC protein is encoded by the third ORF of the ARV S1 segment [14]. It is an elongated trimeric minor outer capsid protein and is responsible for cell attachment [20, 21]. In previous studies, the ARV σC induced high levels of type-specific neutralization antibodies, and the attachment of ARV to permissive cells can be inhibited by pretreatment using recombinant σC protein expressed in *Escherichia coli*, demonstrating an important role of σC as a receptor binding protein [22, 23]. Junctional adhesion molecule-A (JAM-A), which interacts with σ1, a functional and structural homolog of fusogenic orthoreovirus σC proteins, has been identified as a serotype-independent receptor for MRVs [24]. Although the cellular receptor for ARV σC has not been identified, structural studies indicated that the C-terminal globular head domain of σC protein, with a similar overall topology compared to that of MRV σ1, is involved in receptor binding [25]. The third ORF of the S1 segment of NBV is predicted to encode the homologous cell attachment protein σC when compared with the sequences of other reovirus cell attachment proteins [16]. However, the precise functions of NBV σC in the viral life cycle are poorly defined.

A reverse genetics system to engineer viable viruses that contain specific sequence modification is a powerful approach for studying viral replication and pathogenesis and developing vaccines and viral vectors. Although the development of a reverse genetics system for the *Reoviridae* family has lagged behind that for other RNA virus families because of technical complexities associated with the manipulation of multiple-segmented dsRNA genomes, in recent years, reverse genetics systems have been developed for the genus *Rotavirus*, rotavirus; genus *Orbivirus*, bluetongue virus, African horse sickness virus, and epizootic hemorrhagic disease virus; and genus *Orthoreovirus*, MRV [26–33]. Partial plasmid-based reverse genetics systems that are limited to helper virus-dependent and single-gene modification methods have been developed for rotavirus [27, 30]. However, reverse genetics systems that are helper virus-independent have been established for orbivirus and orthoreovirus. Bluetongue virus, African horse sickness virus, and epizootic hemorrhagic disease virus can be rescued by the transfection of *in vitro*-transcribed RNAs into permissive cell lines [29, 31–33]. An entirely plasmid only-based reverse genetics system has been developed for MRV, and viable viruses can be recovered from cloned cDNAs of each of the 10 viral gene segments [28, 34]. More recently, a plasmid only-based system has been established for bluetongue virus [35]. In contrast to reverse genetics systems established for nonfusogenic MRVs, the reverse genetics approach has not been developed for fusogenic orthoreoviruses. This technological issue is perhaps the single most important limitation to studies of the aforementioned fusogenic orthoreoviruses.

In this study, we developed an entirely plasmid-based reverse genetics system for NBV associated with acute upper respiratory tract infection in humans. This is the first genetic manipulation for the fusogenic subgroup of orthoreoviruses using a strategy that does not require a helper virus and selection system. Deletions and point mutations introduced into viral minor outer capsid σC protein were used to define the function of this protein in the viral life cycle. We found that NBV σC is not required for propagation in murine fibroblast L929 cells, but it plays a critical role in the attachment to human lung epithelial A549 cells. We ascertained that the C-terminal globular head domain of the σC protein is involved in binding selectively to the surface of A549 cells and that σC is a determinant of viral virulence in infected mice. We generated a recombinant virus that expresses a yellow fluorescent protein (ZsYellow) by replacement of the σC ORF with ZsYellow. The reverse genetics system can be employed for studies on the replication and pathogenesis of this important group of pathogenic orthoreoviruses.

## Results

### Generation of recombinant NBV from cloned cDNA

**Fig 1:**
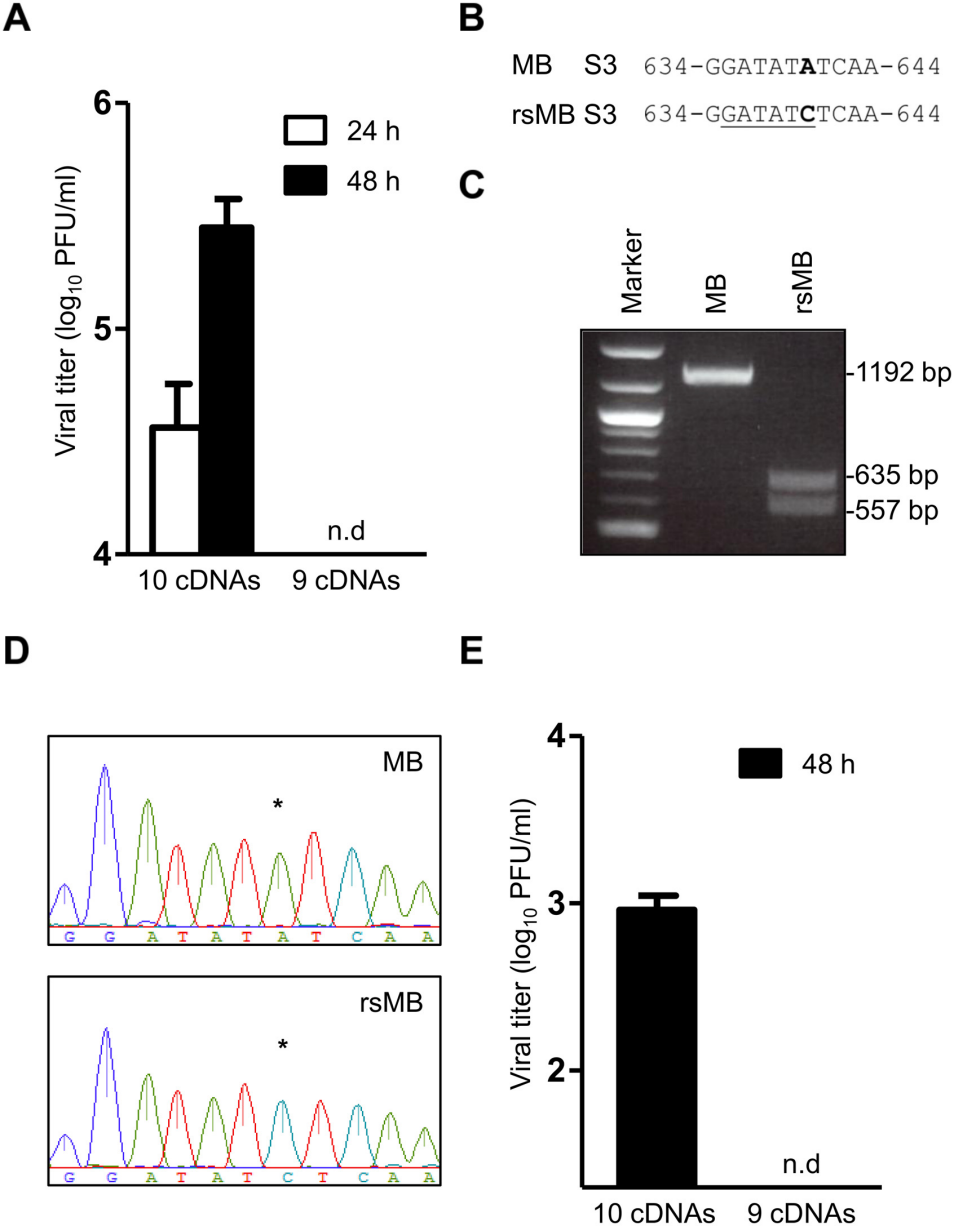
Establishment of reverse genetics system for NBV strain MB. (A) Viral titers in cell lysate following plasmid transfection. L929 cells were infected with rDIs-T7pol for 1 h and cotransfected with 10 plasmids encoding each of the 10 gene segments of strain MB or nine plasmids that did not include a plasmid encoding the S3 segment. The cells were collected at 24 and 48 h post transfection. The viral titers were determined via a plaque assay. Results are represented as the mean for triplicate samples. Error bars indicate standard deviations. n.d: not detected. (B) rsMB contains a signature mutation in the S3 segment as a genetic marker. A nucleotide substitution from A to C was introduced at position 640 to create the EcoRV site in the S3 segment, which is underlined. (C) Digestion of the amplified S3 gene fragments of MB and rsMB using EcoRV to confirm the introduction of a silent point mutation. Size markers are indicated. (D) Sequence analysis of S3 gene fragments from recombinant viruses. The S3 gene fragment was amplified using RT-PCR with viral dsRNA extracted from the purified native MB and rsMB virions. The amplified fragments were subjected to direct sequence analysis. The sequence chromatograms show the A to C substitution at position 640 of rsMB. (E) BHK/T7-9 cells were cotransfected with the 10 MB plasmids. Following 48 h of incubation, viral titers from transfected cells were determined using a plaque assay. Results are presented as the mean for triplicate samples, and error bars indicate standard deviations. n.d: not detected.

To generate the recombinant strain (rs) MB from cloned cDNA, L929 cells were infected with the attenuated vaccinia virus (rDIs-T7pol), which expresses T7 RNA polymerase, 1 h prior to transfection with plasmids encoding cDNAs corresponding to each of the 10 viral gene segments. Each plasmid contains a full-length MB gene-segment cDNA flanked by the T7 RNA polymerase promoter and the hepatitis delta virus (HDV) ribozyme sequences. Transcription using T7 RNA polymerase generates nascent transcripts corresponding to viral full-length (+)-sense RNAs containing the native viral 5′-end. Self-cleavage by the HDV ribozyme generates the native viral 3′-end. The vaccinia virus rDIs-T7pol employed for rescue experiments replicates permissively in chick embryo fibroblasts (CEF), but is incapable of replicating in most mammalian cells [36]. Following incubation, the cells were collected at 24 and 48 h post transfection, and the viral titers were determined by a plaque assay using L929 cell monolayers. Viral titers following transfection with the 10 MB plasmids were 3.6 × 10^4^ and 2.8 × 10^5^ plaque-forming units (PFU)/ml at 24 and 48 h, respectively, whereas recombinant viruses were not rescued from cells transfected with nine MB plasmids that did not include a plasmid encoding S3 segment (Fig 1A). To confirm whether the rescued virus was generated using the cloned cDNA, a unique EcoRV site was created in the S3 segment by the introduction of a silent point mutation, A to C, at nucleotide position 640 in the S3 plasmid (Fig 1B). The viral dsRNA was extracted from MB and rsMB virions. The full-length S3 segment (1192 bp) was amplified using RT-PCR. The amplified S3 gene fragment derived from MB was not digested using EcoRV, whereas the rsMB S3 gene fragment was digested to produce 635- and 557-bp fragments (Fig 1C). The sequence was determined by direct sequencing of the PCR fragment, and the sequence analysis confirmed the expected A to C substitution as a genetic marker in the S3 segment of rsMB (Fig 1D). These results indicate that rsMB originated from the cloned cDNA. To develop a vaccinia virus-free reverse genetics system, we transfected BHK/T7-9 cells stably expressing T7 RNA polymerase with the 10 MB plasmids and determined viral titers. Viral titers following transfection were 9.1 × 10^2^ PFU/ml at 48 h (Fig 1E). These results demonstrate that BHK/T7-9 cells can be used as an alternative to L929 cells infected with rDIs-T7pol for entirely rescuing the infectious virus from cloned DNA.

### Characterization of rsMB generated by reverse genetics

**Fig 2:**
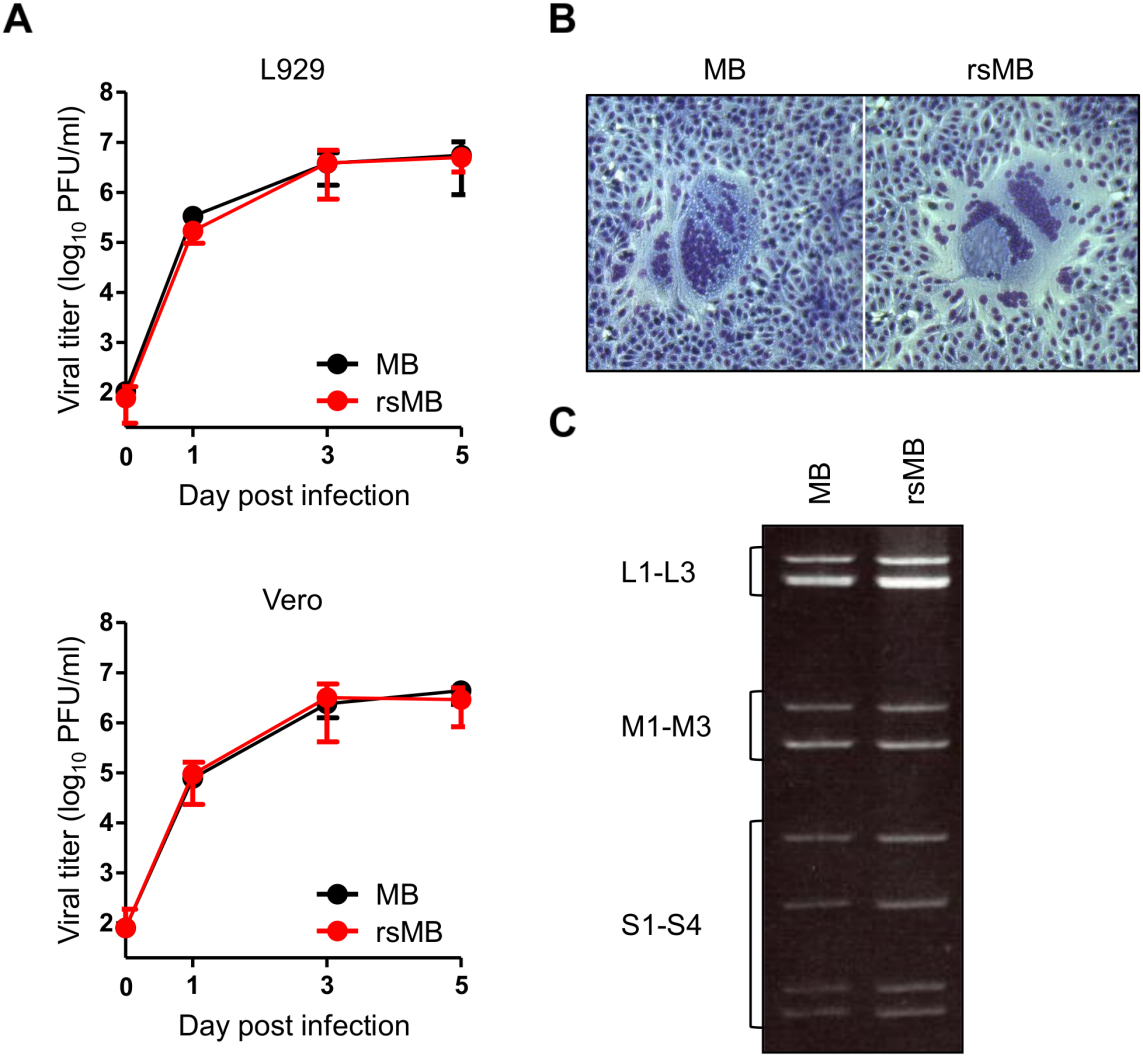
Characteristics of rsMB generated using reverse genetics. (A) Growth kinetics of MB and rsMB in L929 and Vero cells. Cells were infected with recombinant viruses at an MOI of 0.1 PFU/cell and incubated for the intervals shown. Viral titers were determined using a plaque assay. (B) Syncytium formation in Vero cells infected with MB and rsMB. The cells were infected with the viruses and incubated for 12 h. The fixed cells were stained with Giemsa solution to visualize syncytium formation. (C) The electropherotype of dsRNA of MB and rsMB. The viral dsRNA was extracted from purified virions, separated using SDS-polyacrylamide gel electrophoresis, and visualized by ethidium bromide staining. Classes of gene segments based on their sizes (L, M, and S gene segments) are indicated.

To confirm whether rsMB generated from cloned cDNA reflects the characteristics of the parent MB strain, we first investigated the replication kinetics of MB and rsMB in L929 and monkey kidney epithelial Vero cells. Growth kinetics for the two viruses was virtually identical at all the time points (Fig 2A). The fusogenic NBV strain MB exhibits large syncytium formation in infected cells based on the fusogenic ability of FAST protein encoded by the S1 segment [11, 17]. To determine whether rsMB forms a syncytium in a manner similar to native MB, cells were infected with MB and rsMB and processed 12 h post infection for image analysis by Giemsa staining. Both MB and rsMB formed morphologically indistinguishable large syncytia in infected Vero cells (Fig 2B). Furthermore, to confirm that rsMB contains the correct pattern of gene segments, the viral genomic dsRNAs were resolved using sodium dodecyl sulfate (SDS)-polyacrylamide gel electrophoresis. The electropherotype of rsMB was indistinguishable from that of MB (Fig 2C). Collectively, these results demonstrate that the replication characteristics of rsMB are indistinguishable from those of native MB.

### Generation of S1 monoreassortant viruses

**Fig 3:**
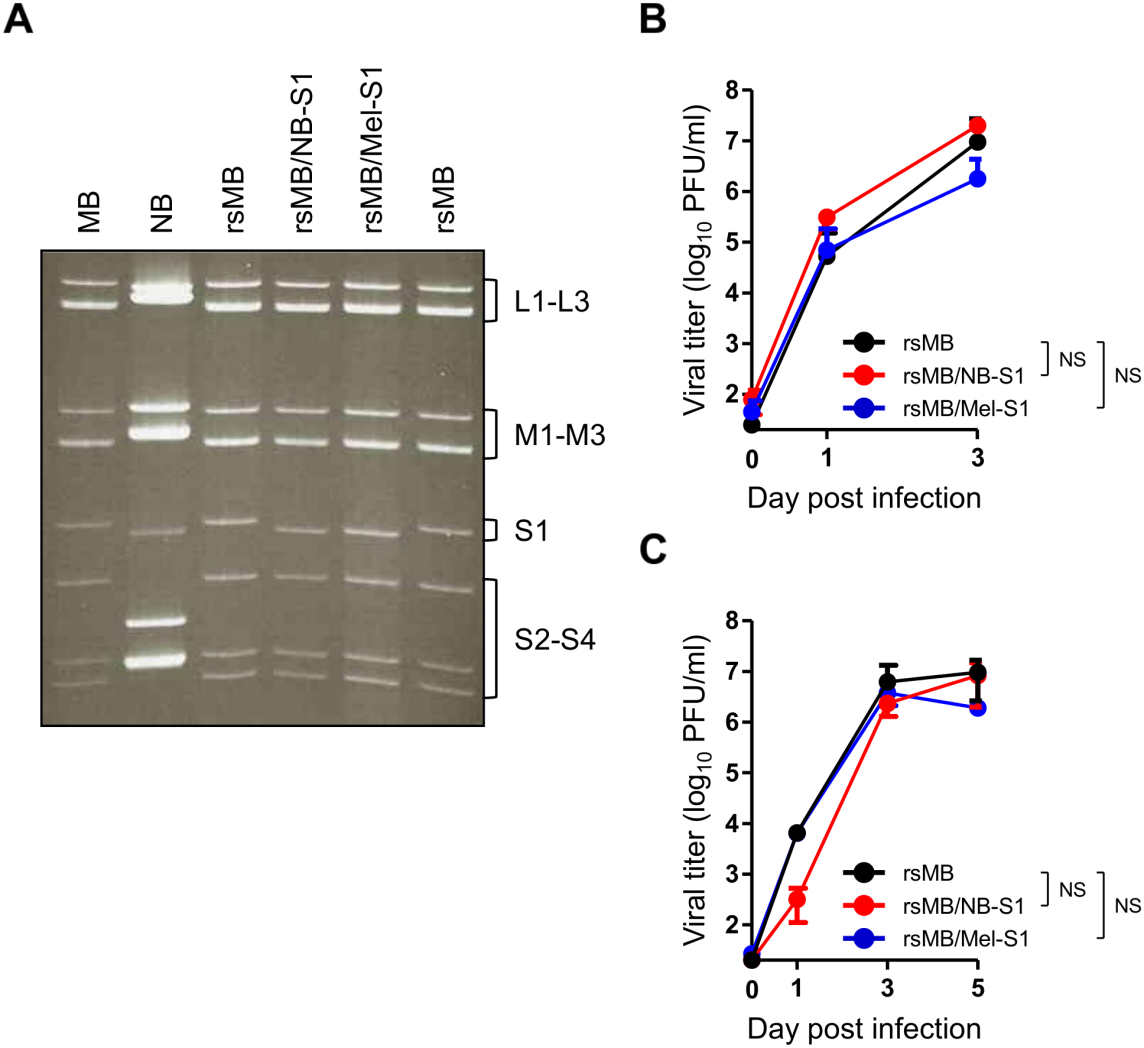
Generation of S1 monoreassortant viruses. (A) Electropherotype of dsRNA of rsMB/NB-S1 and rsMB/Mel-S1 compared with that of the wild-type viruses. The viral dsRNA was extracted from purified virion, electrophoresed, and visualized by ethidium bromide staining. Classes of gene segments based on their sizes are indicated. Growth kinetics of rsMB, rsMB/NB-S1, and rsMB/Mel-S1 in L929 cells. The cells were infected with the recombinant viruses at an MOI of 0.1 (B) or 0.01 (C) PFU/cell and were incubated for various intervals. After freeze-thawing, the viral titer in the cell lysate was determined by a plaque assay. Significant differences in comparison to rsMB were identified using Student’s *t*-test at 3 days post infection for an MOI of 0.1 PFU/cell and at 5 days post infection for an MOI of 0.01 PFU/cell. NS: not significant.

Gene segment reassortment can occur among orthoreovirus species with segmented genomes during coinfection of cells with different strains [37–40]. This event can result in the generation of new viruses with altered virulence. However, the NBV assembly process has not yet been fully resolved and the genome reassortment among NBV strains occurring in nature has not been elucidated. To assess whether genome reassortment can occur among NBV strains, we attempted to generate recombinant monoreassortant viruses, which possess the S1 segment of strains NB or Mel and the other nine segments of strain MB. To rescue S1 monoreassortant viruses, the S1 plasmid, which encodes the full-length S1 segment of both the strains NB and Mel, was cotransfected into rDIs-T7pol-infected L929 cells with other nine plasmids from strain MB, and the viruses were rescued using a plaque assay 48 h post transfection. The electrophoretic pattern of the monoreassortant rsMB/NB-S1, which contains the NB S1 segment in an otherwise MB background, clearly reveals the comigration of S1 RNA with native NB (Fig 3A). The pattern of the monoreassortant rsMB/Mel-S1, which contains the Mel S1 segment in an otherwise MB background, reveals a different electropherotype of S1 RNA with native MB and rsMB (Fig 3A). To assess the replication kinetics of the reassortant viruses in L929 cells, the cells were infected with rsMB, rsMB/NB-S1, or rsMB/Mel-S1 at a multiplicity of infection (MOI) of 0.1 or 0.01 PFU/cell. The recombinant monoreassortant viruses exhibit replication kinetics similar to the native rsMB (Fig 3B and 3C). These results suggest that genome reassortment events can occur among NBV species when the cells are coinfected with different NBV strains, and the recovery of S1 monoreassortant viruses shows the utility of the rescue system to potentially generate reassortant viruses with any desired genetic combination between different NBV strains.

### Recovery of recombinant viruses that lack σC protein expression

**Fig 4:**
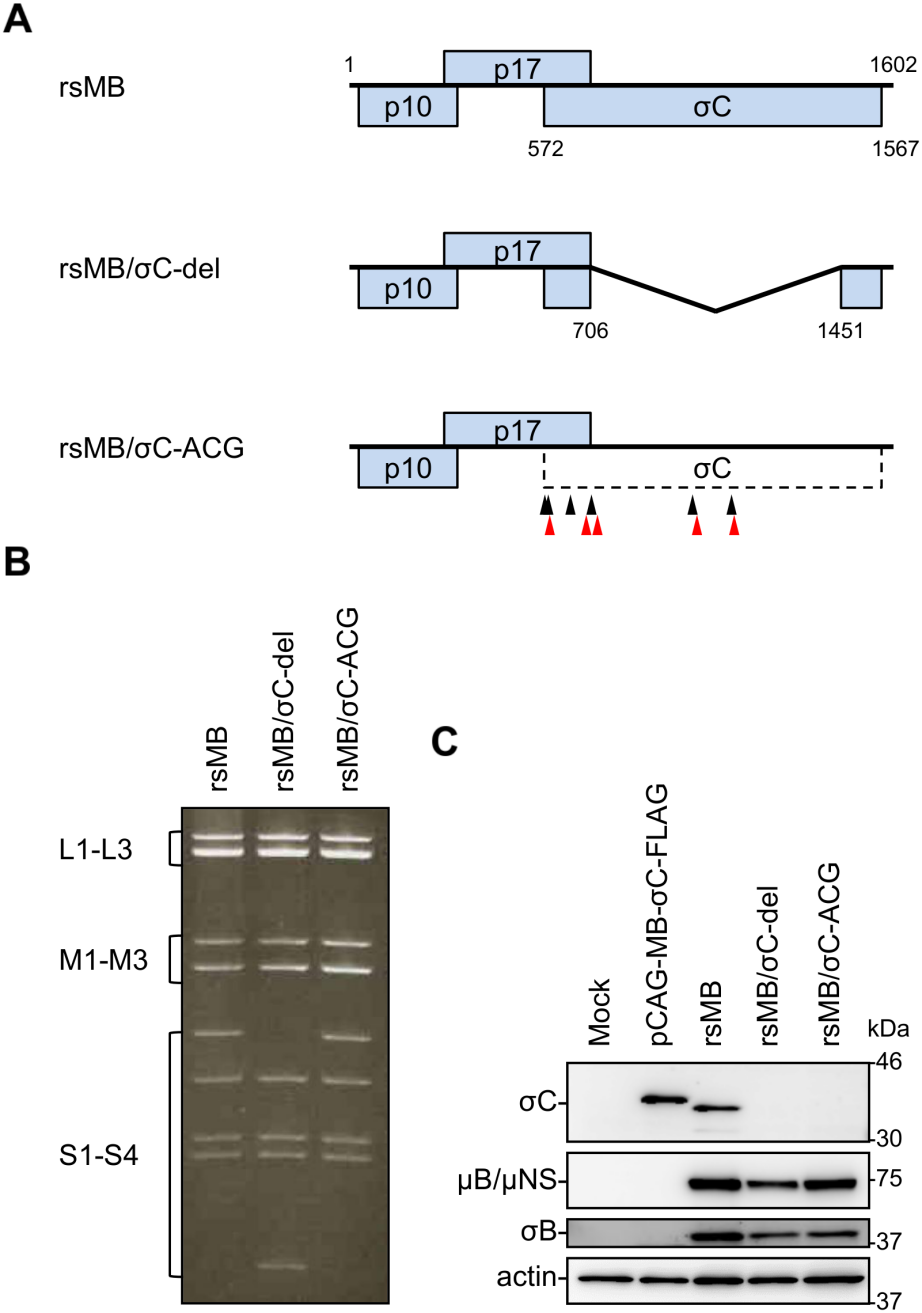
Recovery of recombinant viruses incapable of expressing σC protein. (A) Schematic presentation of the plasmids encoding S1 segment used for the recovery of wild-type, rsMB/σC-del, and rsMB/σC-ACG viruses (pT7-S1MB, pT7-S1MB-σC-del, and pT7-S1MB-σC-ACG, respectively). The plasmid pT7-S1MB-σC-del lacks most of the nucleotide sequence in the ORF of σC. To generate the plasmid pT7-S1MB-σC-ACG, the start codon and other five AUG codons in the σC ORF were disrupted, and other five stop codons were inserted into the σC ORF. The black arrowheads indicate the ACG mutation sites, and the red arrowheads indicate the stop codon mutation sites. (B) The electropherotype of the dsRNA of rsMB, rsMB/σC-del, and rsMB/σC-ACG. The viral dsRNA was extracted from purified virions, electrophoresed, and visualized by ethidium bromide staining. Classes of gene segments based on their sizes are indicated. (C) Expression of σC and other NBV proteins in cells infected with the viruses. L929 cells were transfected with pCAG-MB-σC-FLAG or infected with rsMB, rsMB/σC-del, or rsMB/σC-ACG at an MOI of 0.1 PFU/cell and incubated for 48 h. The cell lysates were analyzed by immunoblotting using σC-specific polyclonal antiserum, NBV-specific antiserum, or antibody specific for actin. The molecular weights of the proteins are shown in kilodaltons (kDa).

In most orthoreoviruses, the S1 segment is functionally polycistronic and encodes σ1/σC, an outer fiber protein of the virions responsible for attachment to the host cell membrane [20, 21, 41]. Although sequence analysis of NBV polycistronic S1 segments revealed that σC is encoded in the third ORF of the S1 segment [16], structural and functional analysis of NBV σC in viral replication has not been performed. In addition, unlike other orthoreoviruses, BRV and BroV isolated from the Australian fruit bat, which are divergent from the NBV species, were found to lack a σ1/σC homolog in their polycistronic S class gene segments [3, 42]. These reports suggest that BRV and BroV may utilize distinct strategies to infect cells compared to those employed by other orthoreoviruses. To define the importance of NBV σC in the viral life cycle, we used a reverse genetics system to generate recombinant viruses incapable of expressing σC protein. Viable σC-null viruses were rescued from cells transfected with cDNAs of nine gene segments and S1 cDNA featuring deletion of most of the nucleotide sequence spanning 707–1450 corresponding to the σC ORF or disruption/insertion of the σC translational start codon/stop codon (rsMB/σC-del and rsMB/σC-ACG, respectively; Fig 4A). Electropherotype analysis of the rescued viruses demonstrated that rsMB/σC-del displayed the expected migration of the S1 segment compared with that of rsMB (Fig 4B). The mutations in the σC ORF of the S1 segment from rsMB/σC-del and rsMB/σC-ACG were confirmed by direct sequencing of viral genomic RNA. To confirm that defective mutations in the σC ORF of the S1 segment from the rsMB/σC-del and rsMB/σC-ACG viruses prevent σC protein synthesis, we assessed the expression of σC protein by immunoblotting with σC-specific antiserum in L929 cells infected with the recombinant viruses. The expression of σC protein was detected in cells infected with the wild-type virus and transfected with FLAG**-**tagged fusion σC protein expression plasmid but not in cells infected with rsMB/σC-del and rsMB/σC-ACG viruses (Fig 4C). Immunoblotting with NBV-specific antiserum demonstrated equivalent expression levels of other NBV proteins (μB/μNS and σB) in cells infected with the wild-type and σC expression-defective viruses, suggesting a similar level of infection by the wild-type and σC-deficient viruses (Fig 4C). We also confirmed that σC proteins were included in purified wild-type virions (S1A Fig). To test the generalizability of virus recovery in the absence of σC protein expression among other NBV strains, we attempted to generate monoreassortant viruses containing the NB or Mel S1 segments lacking σC protein expression in the genetic background of strain MB (S1B Fig). The expected alteration in the S1 segment from each monoreassortant virus was confirmed by electrophoretic mobility using viral dsRNA (S1C Fig). Based on these results, we conclude that recombinant NBVs that lack σC protein can be rescued by reverse genetics as a strain-independent generalizability.

### NBV σC protein is not required for viral replication and apoptosis induction in L929 cells

**Fig 5:**
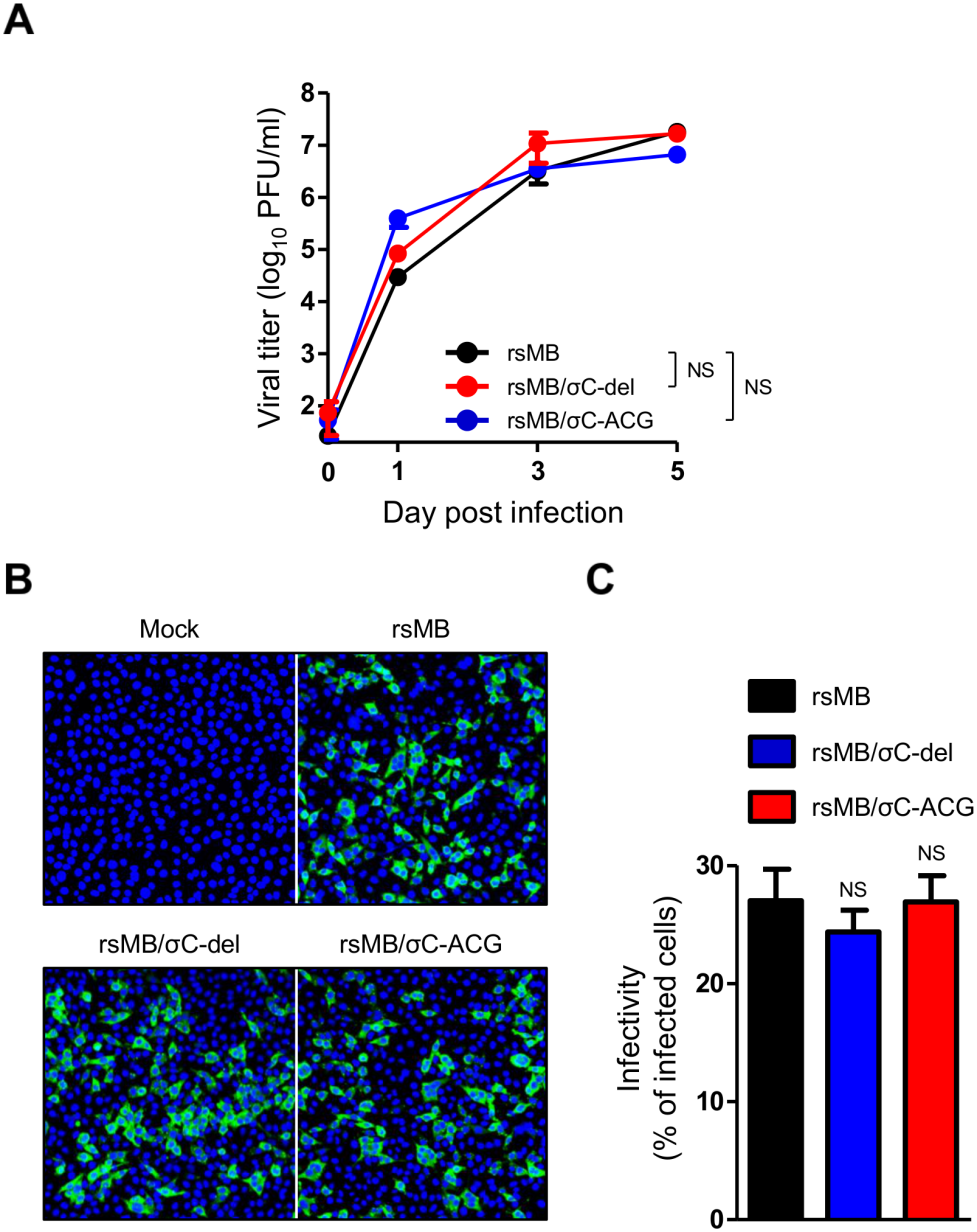
NBV σC is not required for viral replication in L929 cells. (A) Growth kinetics of the rsMB, rsMB/σC-del, and rsMB/σC-ACG in L929 cells. The cells were infected with recombinant viruses at an MOI of 0.1 PFU/cell and incubated for various intervals. After freeze-thawing, the viral titer in the cell lysate was determined using a plaque assay. Significant differences in comparison to rsMB were identified using Student’s *t*-test at 5 days post infection. NS: not significant. (B) The infectivity of rsMB, rsMB/σC-del, and rsMB/σC-ACG in L929 cells. The cells were infected with the viruses at an MOI of 30 PFU/cell and incubated for 12 h. After incubation, the infectivity of the viruses was analyzed by an indirect immunofluorescence assay using NBV-specific polyclonal antiserum. (C) The infectivity rate was calculated as the ratio of the number of infected cells to the total cell population in the image. Results are expressed as the mean infectivity rate for three fields of view. The error bars indicate standard deviations. Significant differences in comparison to rsMB were identified using one-way ANOVA. NS: not significant.

To determine whether σC protein influences NBV growth in cell culture, the viral titers of rsMB/σC-del and rsMB/σC-ACG were determined following infection of L929 cells at an MOI of 0.1 PFU/cell. The replication kinetics and yields of infectious progeny for the rsMB/σC-del and rsMB/σC-ACG viruses were indistinguishable from those of the wild-type viruses (Fig 5A). To further analyze the characteristics of σC-deficient viruses, the infectivity of these viruses to L929 cells was determined using an indirect immunofluorescence assay. The cells were infected with wild-type, rsMB/σC-del, or rsMB/σC-ACG viruses at an MOI of 30 PFU/cell. At 12 h post infection, the infected cells were detected using NBV-specific antiserum. The infectivity did not differ significantly among wild-type, rsMB/σC-del, and rsMB/σC-ACG viruses (27.0%, 24.3%, and 26.9%, respectively), indicating that σC protein is not indispensable for viral replication and infectivity in L929 cells (Fig 5B and 5C). Previous studies have shown that ARV σC and MRV σ1 proteins play roles in regulating apoptosis in infected and transfected cells [43–46]. Therefore, to investigate the role of NBV σC in apoptosis, L929 cells were infected with wild-type and σC-deficient viruses, and apoptosis was quantitated using a caspase 3/7 activity assay (S2 Fig). Wild-type and σC-deficient viruses induced equivalent levels of apoptosis in comparison with those of mock and MRV strain rsT3D [28], which was used as a positive control for this assay (S2 Fig). This result indicates that NBV can induce apoptosis, but σC protein is not required for apoptosis induction in L929 cells.

### NBV σC protein is required for infection in A549 cells

**Fig 6:**
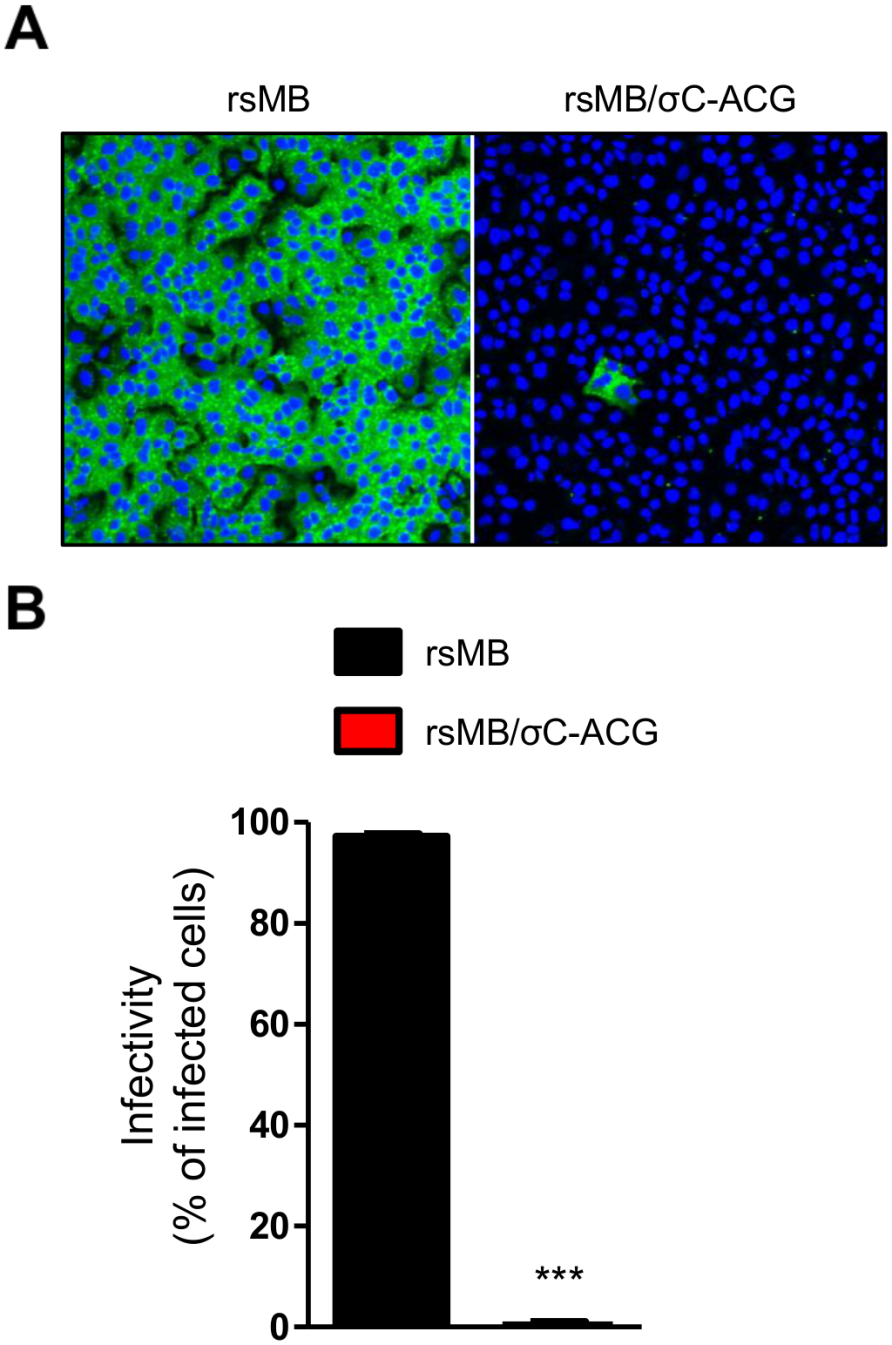
NBV σC is required for infection in A549 cells. (A) Infectivity of rsMB and rsMB/σC-ACG in A549 cells. The cells were infected with the recombinant viruses at an MOI of 10 PFU/cell and incubated for 6 h. Representative images are shown. (B) The infectivity rate was calculated from the ratio of the number of infected cells to number of nucleus in the image. Results are expressed as the mean infectivity rate for three fields of view. The error bars indicate standard deviations. The significant difference was determined using Student’s *t*-test in comparison to rsMB. ***p < 0.0005.

**Fig 7:**
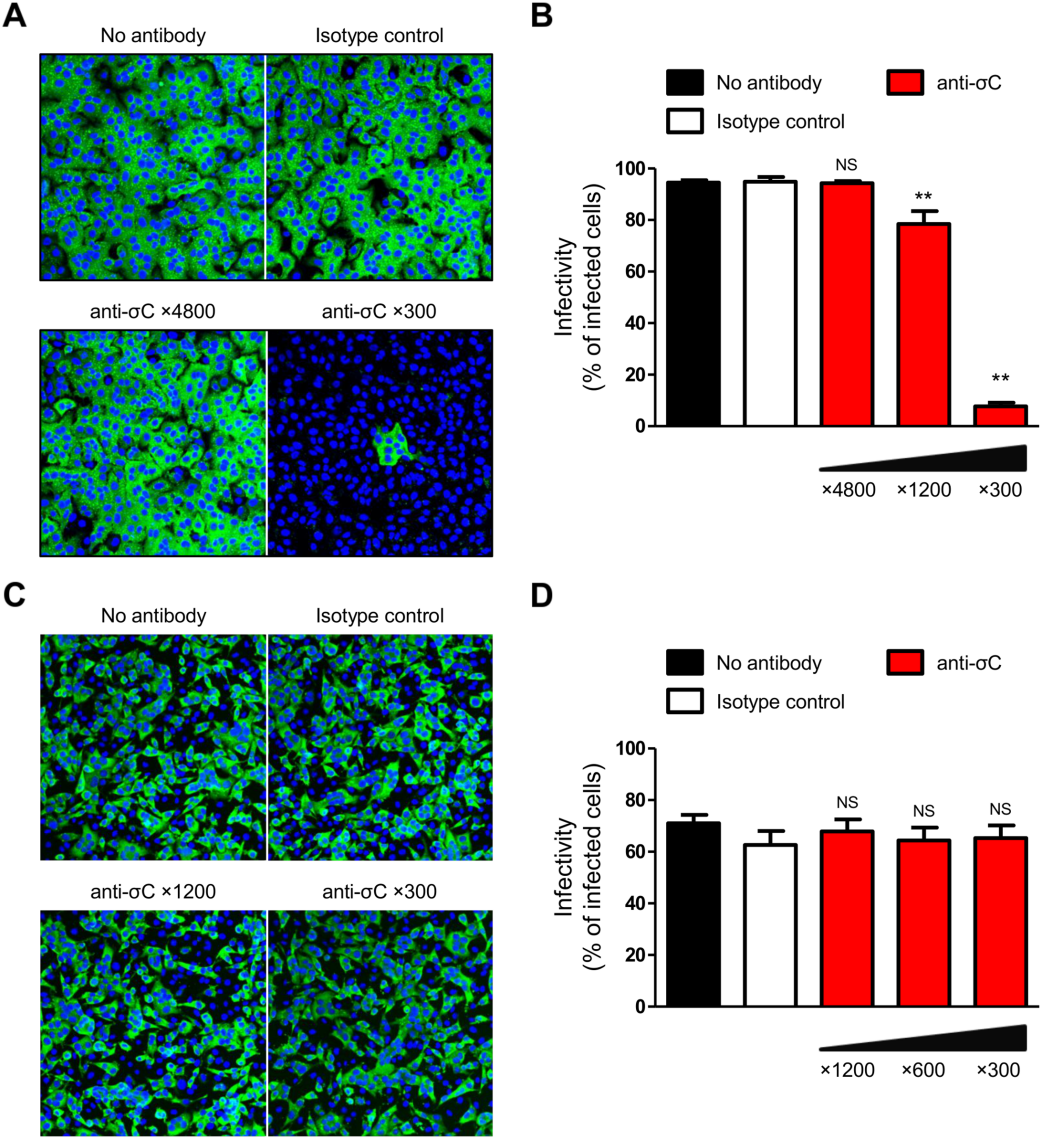
Infection by the wild-type virus is inhibited by treatment using σC-specific antiserum in A549 cells. Viruses were incubated with σC-specific antiserum for 1 h and used to infect A549 and L929 cells at MOIs of 30 and 10 PFU/cell, respectively. After incubation for 6–12 h, the infectivity of the viruses in A549 (A) and L929 cells (C) was analyzed by an indirect immunofluorescence assay using NBV-specific antiserum. The infectivity rate in A549 (B) and L929 cells (D) was calculated as the ratio of the number of infected cells to the total cell population in the image. Results are expressed as the mean infectivity of three fields of view. The error bars indicate standard deviations. Significant differences in comparison to the isotype control were identified using one-way ANOVA. NS: not significant; **p < 0.005.

**Fig 8:**
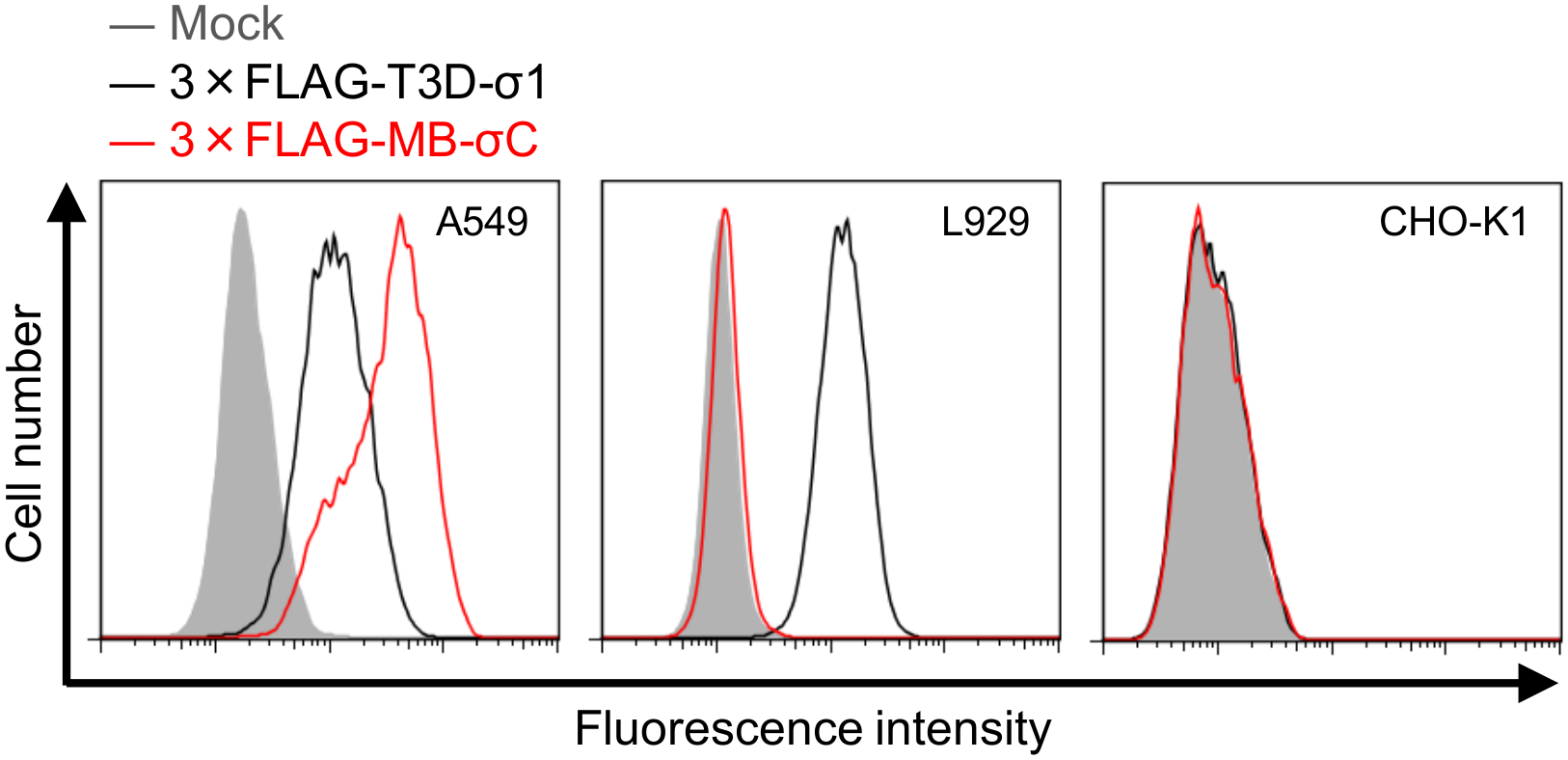
NBV σC binds to the surface of A549 cells. Binding capacity of 3 × FLAG-MB-σC and 3 × FLAG-T3D-σ1 in culture cell lines. A549, L929, and CHO-K1 cells were incubated with the recombinant soluble proteins for 1 h, and the number of cells bound by the protein was quantified by flow cytometry.

If NBV can bind to cell-surface receptors through σC, cell lines with significantly reduced susceptibility to infection by σC-deficient viruses may exist. To test this hypothesis, several cell lines were infected with the σC-deficient viruses and viral infectivity was assessed by an indirect immunofluorescence assay using NBV-specific antiserum. We found that the infectivity of rsMB/σC-ACG markedly decreased in comparison to that of the wild-type virus in A549 cells (Fig 6A and 6B). Following infection of other cell lines, such as hamster kidney fibroblast BHK-21, hamster ovary CHO-K1, bat kidney DemKT1 [47], and Vero cells, the viral infectivity of rsMB/σC-ACG and wild-type viruses was not found to differ (S3 Fig). To further investigate whether σC plays a critical role in viral infectivity, we determined whether σC-specific antiserum could inhibit NBV infection in A549 cells. The wild-type virus was treated using σC-specific antiserum at various concentrations prior to infection. The number of infected A549 cells significantly decreased in a concentration-dependent manner (Fig 7A and 7B). In contrast, the wild-type virus efficiently infected L929 cells, which are susceptible to wild-type and σC-deficient viruses, regardless of the presence or absence of σC-specific antiserum (Fig 7C and 7D). To determine whether wild-type and rsMB/σC-ACG viruses can bind to A549 cells, A549 cells were incubated with wild-type or rsMB/σC-ACG virions and scored for virus binding using flow cytometry. Wild-type virions bound to A549 cells, but rsMB/σC-ACG virions were incapable of binding to A549 cells (S4A Fig). When wild-type virus was treated with σC-specific antiserum prior to infection, the amount of virus binding was significantly inhibited (S4A Fig). To further investigate the importance of σC protein during the infection of A549 cells, we performed a cell-surface binding assay using soluble σC protein with 3 × FLAG-tagged epitopes (3 × FLAG-MB-σC). Recombinant soluble MRV strain T3D σ1 protein (3 × FLAG-T3D-σ1), which is required for cell attachment of the MRV infection, was used as a positive control for the binding assay. Immunoblotting using FLAG antibody demonstrated that both proteins were expressed and purified from the soluble fraction (S4B Fig). Binding of soluble σ1 was observed for A549 and L929 cells but not for CHO-K1 cells, which reflects the cellular tropism of MRVs (Fig 8). When the A549 cells were incubated with 3 × FLAG-MB-σC, the fluorescence intensity increased compared with that of mock-treated cells (Fig 8). In contrast, binding of σC protein was not observed for L929 and CHO-K1 cells (Fig 8). In addition, binding of σC protein was not observed for other cell lines such as BHK-21, DemKT1, and Vero cells (S4C Fig). These results provide evidence that σC protein is required for efficient infection in A549 cells and that this protein plays an important role in viral attachment to host cells.

### Identification of the responsible region in σC protein for cell attachment

**Fig 9:**
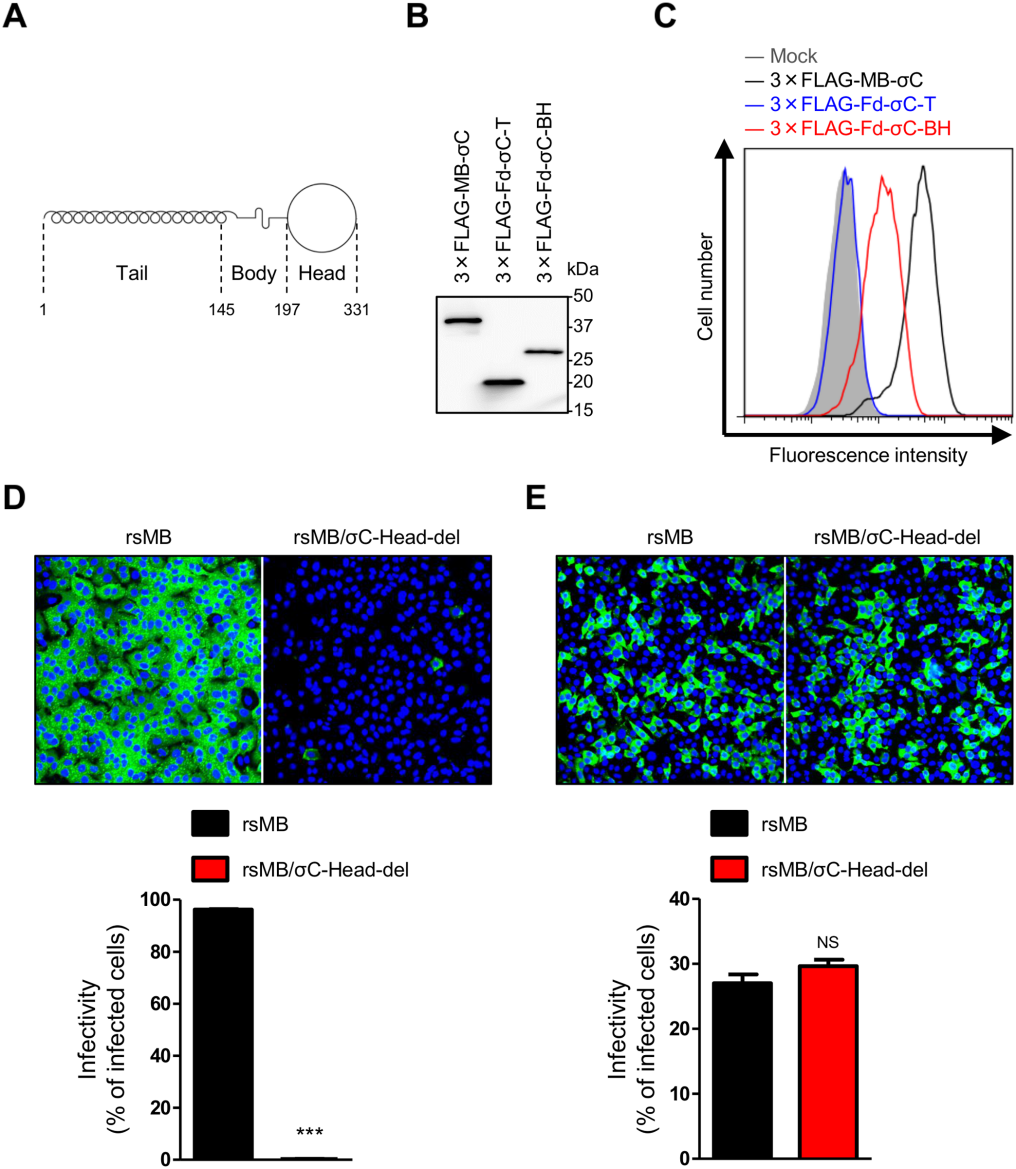
The C-terminal region of σC is required for cell attachment and infection in A549 cells. (A) Schematic presentation of σC protein structure as predicted by SWISS-MODEL (http://swissmodel.expasy.org/). NBV σC comprises tail, body, and head regions. (B) Expression and purification of 3 × FLAG-MB-σC, 3 × FLAG-Fd-σC-T, and 3 × FLAG-Fd-σC-BH proteins. 293T cells were transfected with p3×FLAG-MB-σC, p3×FLAG-Fd-σC-T, or p3×FLAG-Fd-σC-BH using 1 mg/ml polyethyleneimine solution. After purification of the recombinant proteins from the cell lysate, the proteins were analyzed by immunoblotting using anti-FLAG-M2 antibody. The molecular weights of the proteins are shown in kilodaltons (kDa). (C) The binding capacity of soluble truncated σC proteins to A549 cells. The cells were incubated with soluble σC proteins for 1 h, and the number of cells bound by the proteins was quantified by flow cytometry. The infectivity of the wild-type and rsMB/σC-Head-del viruses in A549 (D) and L929 cells (E). The viruses were used to infect A549 and L929 cells at MOIs of 10 and 30 PFU/cell, respectively. The infectivity of the viruses was analyzed by an indirect immunofluorescence assay using NBV-specific antiserum. Representative images are shown (upper). The infectivity rate was calculated as the ratio of the number of infected cells to the total cell population in the image (lower). Results are expressed as the mean infectivity of three fields of view. The error bars indicate standard deviations. Significant differences in comparison to rsMB were identified using Student’s *t*-test. NS: not significant; ***p < 0.0005.

Orthoreovirus cell attachment proteins (ARV σC and MRV σ1) form an elongated homotrimeric fiber topped with a globular head [23, 48, 49]. The C-terminal head domain of cell attachment proteins has the same topology in both viruses and plays a key role in receptor recognition [24, 25, 50–52]. Although an interaction between the ARV receptor and the C-terminal head domain has not yet been identified, the MRV receptor has been identified as JAM-A [24]. Automated comparative protein structure modeling using SWISS-MODEL (http://swissmodel.expasy.org/) showed that the NBV σC is composed of three domains, the N-terminal tail, body, and C-terminal head regions, similar to ARV and MRV (Fig 9A). Thus, to define the structural function of NBV σC in cell attachment, truncated σC proteins, namely, 3 × FLAG-Fd-σC-T encoding amino acid residues 1–145 corresponding to the predicted tail domain and 3 × FLAG-Fd-σC-BH encoding amino acid residues 146–331 corresponding to the predicted body and head domains, were purified from transfected cells. The foldon sequence of T4 phage fibritin was inserted at the N-terminus of σC protein to facilitate stabilization of the trimer structure [53]. Expression of truncated σC protein was confirmed by immunoblotting using σC-specific antiserum (Fig 9B). When A549 cells were incubated with soluble 3 × FLAG-Fd-σC-BH, the fluorescence intensity significantly increased, although the intensity was lower than that of wild-type 3 × FLAG-MB-σC (Fig 9C). In contrast, 3 × FLAG-Fd-σC-T failed to bind A549 cells (Fig 9C). These results suggest that the C-terminal head domain of σC protein participates in cell attachment of A549 cells. To further investigate the role of the head domain of σC protein in viral infection, we used reverse genetics to generate reoviruses expressing truncated σC. A stop codon was inserted into the σC ORF at amino acid position 197 to yield viruses lacking the head domain of σC protein (rsMB/σC-Head-del). The infectivity of rsMB/σC-Head-del was significantly decreased in A549 cells in comparison with that of wild-type (Fig 9D). In contrast, infectivity did not differ significantly in L929 cells between the wild-type and rsMB/σC-Head-del viruses (Fig 9E). Overall, these results suggest that the C-terminal head region of σC protein is required for efficient infection in A549 cells.

### NBV σC protein enhances viral pathogenesis in infected mice

**Fig 10:**
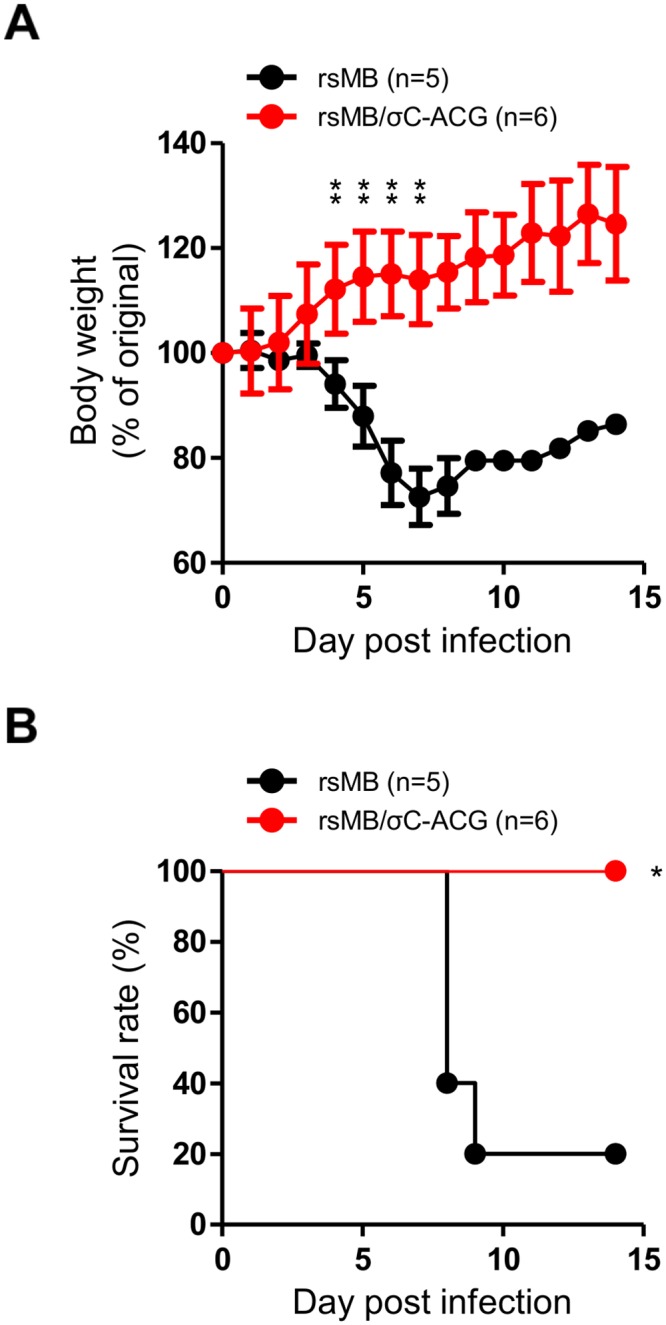
NBV σC is required for pathogenicity *in vivo*. Five or six C3H mice were intranasally infected with 4 × 10^5^ PFU of rsMB or rsMB/σC-ACG, respectively. The mice were monitored for body weight changes (A) and survival rate (B) for 14 days following virus inoculation. (A) Results are expressed as the mean body weight of living mice. The error bars indicate standard deviations. Significant difference in the body weights of mice infected with rsMB/σC-ACG compared to that of mice infected with rsMB was determined using Student’s *t*-test. **p < 0.005. (B) Significant difference in the survival rates of mice infected with rsMB/σC-ACG compared to that of mice infected with rsMB was determined using log rank test. *p < 0.05.

Although σC is not required for viral replication and infectivity in several cell cultures (Fig 5 and S3 Fig), it is possible that σC contributes to viral virulence *in vivo*. Therefore, to determine whether σC influences viral pathogenesis, we inoculated 4-week-old C3H mice intranasally with 4 × 10^5^ PFU of rsMB or rsMB/σC-ACG and assessed the daily disease progression in the mice. A significant decrease in body weight was observed in mice infected with rsMB at 4 to 7 days post infection compared to that of mice infected with rsMB/σC-ACG (Fig 10A). Eighty percent of the mice infected with rsMB died within 14 days post infection (Fig 10B). In contrast, all the mice in the group infected with rsMB/σC-ACG survived (Fig 10B), suggesting that σC plays an important role in viral pathogenesis.

### Generation of a recombinant NBV expressing a foreign gene

**Fig 11:**
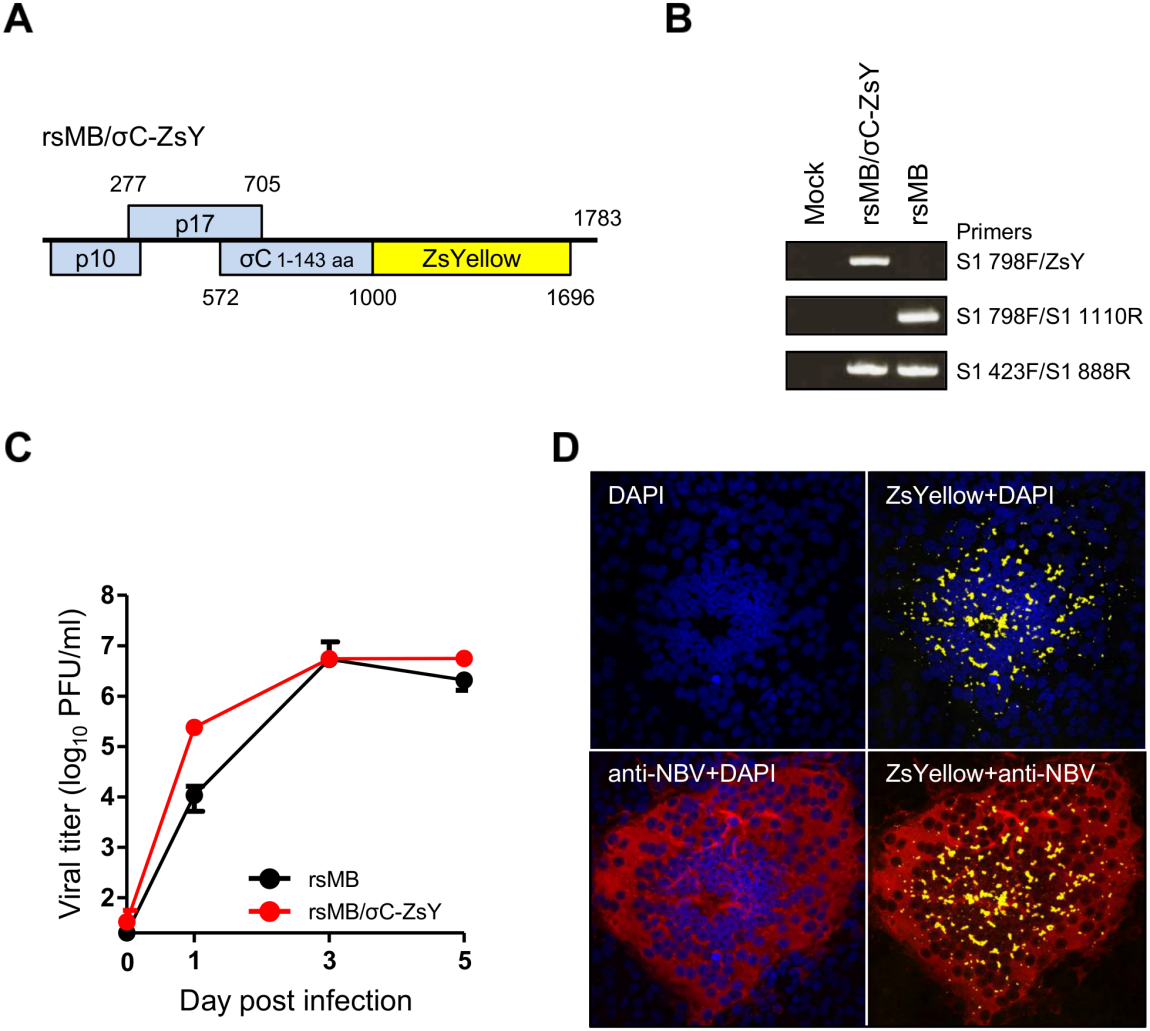
Recovery of a recombinant virus expressing a fluorescence protein. (A) Schematic presentation of the S1 gene segment used for the recovery of rsMB/σC-ZsY (pT7-S1MB-σC-ZsY). The nucleotide sequence of 1001–1516 within the σC gene was replaced with the ZsYellow gene. (B) RT-PCR analysis of rsMB and rsMB/σC-ZsY. The S1 gene fragment was amplified by RT-PCR using viral dsRNA extracted from virions and primers specific for the MB S1 and ZsYellow sequences. The numbers show the S1 nucleotide position corresponding to the 5′ end of the S1-specific primers. (C) Growth kinetics of rsMB and rsMB/σC-ZsY in L929 cells. The cells were infected with the viruses at an MOI of 0.01 PFU/cell and incubated for various intervals. After freeze-thawing, the viral titer was determined by a plaque assay. (D) Expression of ZsYellow in cells infected with rsMB/σC-ZsY. Vero cells were infected with rsMB/σC-ZsY at an MOI of 0.05 PFU/cell and incubated for 24 h. Expression of ZsYellow in the syncytia of Vero cells was observed by confocal microscopy (yellow). Infected cells were fixed and stained using NBV-specific antiserum, followed by Alexa Fluor 633 Goat Anti-Mouse IgG second antibody (red). Cells were stained with DAPI to label nuclei (blue).

The analysis of the σC-deficient viruses demonstrated that σC was not essential for viral replication in L929 cells and led us to postulate that the σC ORF could be replaced by a foreign gene. To test this hypothesis, we introduced sequences encoding ZsYellow into the σC ORF of the MB S1 plasmid (Fig 11A). ZsYellow is expressed as a fusion protein with amino acids 1–143 of σC at the N-terminus. RT-PCR analysis using specific primers for MB S1 and ZsYellow genes confirmed incorporation of a recombinant ZsYellow gene in the S1 gene segment of the resultant virus, rsMB/σC-ZsY (Fig 11B). The replication-competent rsMB/σC-ZsY was capable of replicating in L929 cells, similar to the wild-type virus (Fig 11C). Expression of ZsYellow fused with amino acids 1–143 of σC and NBV antigens was clearly observed in the syncytia of Vero cells infected with rsMB/σC-ZsY (Fig 11D). These results demonstrate that NBV can be engineered to express a foreign gene by replacing the σC ORF.

## Discussion

Although the nonfusogenic MRVs are relatively mild or asymptomatic, the fusogenic orthoreoviruses are pathogenic and cause various severe symptoms in vertebrates [2, 3, 54–56]. Several NBVs have recently been identified as causative agents of severe respiratory tract infections in humans [4, 7–11], suggesting that infections by bat-borne orthoreoviruses may represent potential threats such as emerging zoonosis. To date, no reverse genetics system has been developed for fusogenic orthoreoviruses; therefore, progress in understanding fusogenic orthoreovirus biology and disease has been restricted by this technological barrier. In this study, we established a plasmid-based reverse genetics system for fusogenic NBV strain MB isolated from a patient with acute respiratory infection based on the reverse genetics systems previously developed for MRV [28]. This system permits the selective introduction of desired mutations into the viral genomes using a strategy that does not require a helper virus and selection system. Growth kinetics, cell-to-cell fusion ability, and genomic electrophoretic profiles were indistinguishable between rsMB and native MB (Fig 2). These results demonstrate that the replication characteristics of rsMB generated from cloned cDNA reflect native MB and provide an important advance as a new tool to investigate the molecular biology of NBV propagation and pathogenesis. In previous studies, viral titers following transfection with 10 plasmids encoding the genomes of the MRV strains T1L and T3D were ~10 PFU/ml and below the limit of detection at 24 h post transfection, respectively [28, 34]. In contrast, in the newly developed NBV reverse genetics system, the viral titer (~10,000 PFU/ml) following transfection with the 10 MB plasmids was markedly higher at 24 h post transfection (Fig 1). The peak titer (~10^7^ PFU/ml) of the NBV strain MB is at a similar level or lower in comparison to those of the MRV strains T1L and T3D in L929 cells employed for virus rescue [28, 34]. A potential explanation may be that the NBV strain MB exhibits more efficient replication over the MRV strains during early replication events including dsRNA genome synthesis and core assembly following transfection with NBV cDNA into L929 cells. The reverse genetics system for NBV developed in this study may have a significant advantage to promote the rescue of highly attenuated viruses and viral vectors that have been difficult to recover using current rescue methods for the family *Reoviridae*. In addition, the higher efficiency of the NBV reverse genetics system compared to other *Reoviridae* rescue systems may allow us to progress studies of the common replication pathway shared by the family *Reoviridae*, which is poorly understood including dsRNA genome synthesis, gene segment packaging, and virion assembly. We also developed a vaccinia virus-free reverse genetics system using BHK/T7-9 cells. Although the rescue efficiency of the vaccinia virus-free system was lower than that of the rDIs-T7pol-based system, the elimination of the vaccinia virus makes this reverse genetics system an alternative and is a simple approach for the recovery of recombinant viruses (Fig 1). The reverse genetics for multiple-segmented viruses appears to be inefficient because of the limitation of the transfection efficiency of the target cells. Previous studies have shown that the efficiency of virus rescue using a strategy in which the number of required plasmids was reduced from 10 to 4 was substantially increased in comparison to the first-generation system for the MRV strains T1L and T3D [34]. Thus, further refinement of the rescue system of NBV with a reduction in the total number of plasmids will expand the utility of reverse genetics for studies of NBV biology.

Viruses have an extraordinary ability to adapt and evolve. For segmented viruses including reovirus family viruses, genome reassortment events occur in cells coinfected with more than two different virus strains and play a key role in introducing genomic and phenotypic changes leading to virus diversity. In the genus *Orthoreovirus*, reassortment events have been reported for ARVs and MRVs [37–40]. Although phylogenic analysis based on nucleotide sequences of S class gene segments from different NBV strains suggests the possibility of genome gene reassortment among these viruses, there is no direct evidence whether gene reassortment occurs during mixed infection in culture cells or animal hosts. We used the plasmid-based reverse genetics system to generate monoreassortant viruses containing the S1 segment from strains NB (isolated from bats) or Mel (isolated from humans) in an otherwise MB background. As with the other nine NBV gene segments, S1 segments from strains MB, NB, and Mel possess conserved nucleotide sequences that are identical for the NBV species at the 5′- (5′-GCUU-3′) and 3′-ends (5′-UCAUC-3′); however, the S1 gene segment (σC protein) from strain MB shows significant sequence diversity in terms of nucleotides [66.0% (Mel) and 57.9% (NB)] and amino acid [57.3% (Mel) and 43.6% (NB)], in comparison with the other nine gene segments among these strains isolated from humans and bats [1, 4, 11, 12, 16, 57]. We demonstrated the generation of S1 monoreassortant viruses in experimental culture conditions using the reverse genetics system, suggesting that reassortant events may occur among different NBV strains in nature and that the development of a reverse genetics system may allow us to generate any desired combinatorial exchange for the systematic characterization of gene segments with phenotypic differences among various NBV strains. Genetic experiments using recombinant reassortant viruses will provide key insights into viral replication and pathogenesis. Growth kinetics for the S1 monoreassortant viruses was virtually identical to that of the wild-type viruses (Fig 3). Previous reports using reassortant viruses to understand viral pathogenic mechanisms demonstrate that the orthoreovirus S1 segments influence strain-specific differences in viral replication in tissues and the pathway of spread in the host [39, 58–63]. Thus, it may be possible that monoreassortant viruses containing S1 segments derived from different NBV strains promote distinct viral growth characteristics in certain cell or tissue types to promote spread within or between hosts.

We used the NBV reverse genetics system to introduce mutations in σC protein, which probably forms part of the viral minor outer capsid and is considered essential for virus infection. In other orthoreoviruses, MRV σ1, which is analogous to σC protein, functions as the viral cell attachment protein [41] and is the primary virulence determinant through the cellular receptor binding for cell attachment or through the intracellular signal transduction pathways mediated by receptor binding [64–66]. Conversely, BRV and BroV do not exceptionally encode homologs of the cell attachment proteins σ1 and σC in their polycistronic S gene segments, suggesting that a σ1/σC homolog-independent cell attachment pathway to bind to the cell-surface receptors may also exist in orthoreovirus entry [3, 42]. Therefore, we applied our reverse genetics system to study the σC protein to understand the mechanism by which this protein mediates critical steps in viral replication. The resulting σC-deficient viruses are viable (Fig 4 and S1 Fig), and the caspase 3/7 activity assay using cells infected with wild-type or σC-deficient viruses indicated that the σC protein is not required for apoptosis induction in L929 cells (S2 Fig). The single-cycle replication kinetics and infectivity of σC-deficient viruses indicate that σC protein is dispensable for reovirus propagation in several cell lines including L929 cells (Fig 5 and S3 Fig). However, the virus infectivity of σC-deficient virus was significantly diminished in A549 cells (Fig 6), and pretreatment of a wild-type virus with σC-specific antiserum blocked infection in A549 cells (Fig 7), suggesting that σC functions in viral cell attachment of A549 cells. Flow cytometry confirmed that recombinant soluble σC protein binds the surface of A549 cells, which is nonpermissive to σC-deficient virus infection, but not the surfaces of L929, CHO-K1, BHK-21, DemKT1, or Vero cells, which are permissive to σC-deficient virus infection (Fig 8 and S4 Fig). These results provide direct evidence that NBV uses σC protein for cell attachment and infection in A549 cells and suggest that NBV uses σC and different capsid proteins to bind distinct cell-surface components, engaging independent receptors to facilitate virus infection.

Our findings lead us to question regarding which other NBV proteins function as virus ligands for cell attachment in cell lines that are permissive to σC-deficient virus infection. Based on the structural analysis of ARV and MRV virions assumed to be similar to NBV virions [67, 68], a candidate is the major outer capsid protein σB, which is considered to be present in 600 copies and form 200 heterohexameric complexes with a more internal layer composed of 600 copies of μB protein per virion. Recently, Konopka-Anstadt *et al*. demonstrated that MRV uses the Nogo receptor NgR1 for entry into neurons [69]. Further experiments revealed the possibility that MRV uses σ3, a functional homolog of σB, to attach to NgR1, but more studies are required to identify the binding partner for NgR1 [69]. Another candidate involved in cell attachment for σC-deficient viruses is λC, which probably forms a pentameric turret at the virion fivefold symmetry axes, providing a total of 60 copies per virion and serving as the insertion site for the attachment protein σC [67, 68]. MRV λ2, which is a λC homolog, contains conserved integrin binding sequences, suggesting that λ2 mediates the internalization of MRV to enter cells through an interaction with integrins [70]. However, direct binding between NBV proteins such as λC and integrins has not been reported.

Although there are no reports of cellular receptors for ARV and NBV σC proteins, the MRV receptor JAM-A has been identified as a binding partner of σ1, which is the structural and functional homolog of σC protein, for MRV entry [24]. The C-terminal globular head domain is predicted to play a key role in receptor recognition from previous MRV σ1 studies on cell attachment [24, 50–52]. Therefore, we investigated and demonstrated the importance of the C-terminal head domain of NBV σC in cell attachment of A549 cells (Fig 9). However, NBV σC probably binds to distinct but as yet unknown receptor molecules because recombinant σC protein binding was not observed for L929 cells, which express JAM-A and are susceptible to MRV infection. (Fig 8). Although it is known that the nonfusogenic MRV cell attachment protein σ1 is a major determinant of reovirus disease [58–60, 62], the contribution of fusogenic NBV σC to viral virulence has not been elucidated. In this study, we demonstrated the importance of σC in viral pathogenesis using a mouse model of NBV infection (Fig 10). These results suggest that the cell attachment function of σC may contribute to viral infection and pathogenesis *in vivo*, even though it is not required for cell binding in various cell lines. The alternative usage of receptors for entry by NBVs *in vivo* may be potentially significant. NBVs may use multiple independent viral ligands and cellular receptors to internalize into different cell types, as governed by the expression patterns of the entry receptors. Alternatively, NBVs may use distinct ligands and receptors for entry into the same cell type under different cellular conditions. In addition to the selective engagement of viral receptors by NBVs, post-entry signaling events may contribute to disease pathogenesis. Thus, future studies should focus on understanding the mechanism by which selective receptor recognitions and post-binding signaling pathways of cell attachment proteins contribute to viral replication and pathogenesis.

We generated a replication-competent NBV expressing ZsYellow and demonstrated that the S1 gene segment encoding σC was suitable for the insertion of a foreign gene (Fig 11). A replication-competent NBV expressing a reporter gene will allow novel approaches for the study of NBV replication and pathogenesis both *in vitro* and *in vivo* and will provide a powerful system for examining NBV entry and for the identification of NBV receptors.

We have established a reverse genetics system that allows the recovery of the pathogenic NBV strain MB from cloned cDNA. Recombinant σC mutant viruses provide new insights for understanding the viral entry machinery and orthoreovirus evolution through the gain or loss of cell attachment proteins. Unlike nonfusogenic MRVs, fusogenic orthoreoviruses encode two novel nonstructural viral proteins, FAST and NSP (p16 or p17), in polycistronic S gene segments [2, 3, 15–19, 42, 71, 72]. However, the precise functions of these viral proteins in the viral life cycle are poorly understood. We expect that recombinant NBVs in which FAST, NSP, and other viral proteins are systematically altered using this reverse genetics will provide new insights into the viral replication machinery and establish a platform to advance basic and applied research for the family *Reoviridae*.

## Materials and Methods

### Cells and viruses

A549, CHO-K1, L929, Vero, and human embryonic kidney 293T cells were obtained from the American Type Culture Collection and were grown in Dulbecco’s modified Eagle’s medium (DMEM; Nacalai Tesque) supplemented with 5% fetal bovine serum (FBS; Gibco), 100 units/ml penicillin, and 100 μg/ml streptomycin (Nacalai Tesque). BHK/T7-9 cells, a derivative of BHK cells, were grown in DMEM supplemented with 5% FBS, 10% tryptose phosphate broth, 100 units/ml penicillin, and 100 μg/ml streptomycin [73]. NBV strain MB was isolated from a patient with acute respiratory tract infection in Japan in 2007 [11]. NBV strain NB was isolated from the heart blood of a flying fox collected in Australia in 1968 [5]. Virus titers were determined using a plaque assay with L929 cell monolayers as previously described with slight modification [74]. The attenuated vaccinia virus rDIs-T7pol expressing T7 RNA polymerase was propagated in CEF [36]. CEF cells were prepared from 11-day-old embryonated eggs following standard procedures.

### Sequence determination of MB

The genomic viral dsRNA was extracted from purified virions using Sepasol-RNA I Super reagent (Nacalai Tesque). cDNA corresponding to each MB gene segment were amplified from viral dsRNA via the full-length amplification of cDNA as previously described [75]. Briefly, a self-priming anchor-primer was ligated to the 3′-ends of viral dsRNAs using T4 RNA ligase (Thermo scientific). The adaptor-ligated NBV dsRNAs purified by agarose gel electrophoresis were reverse transcribed using Superscript III reverse transcriptase (Invitrogen). PCR amplification was performed using the KOD-Plus-NEO polymerase (Toyobo) with a single primer complementary to the anchor primer. Amplified full-length viral cDNA was blunt-end ligated into the EcoRV site of pBluescript KS (+) vector. Primer sets were designed on the basis of the nucleotide sequences of the NBV strain Kampar [7, 57]. The viral sequences were determined using the ABI 3130 genetic analyzer (Life Technologies).

### Plasmid construction

To generate the rescue plasmids pT7-L1MB, pT7-L2MB, pT7-L3MB, pT7-M1MB, pT7-M2MB, pT7-M3MB, pT7-S1MB, pT7-S2MB, pT7-S3MB, and pT7-S4MB encoding the full-length cDNA of each gene segment derived from MB, viral cDNA-containing fragments were subcloned into pT7-L1T3D, which encodes the full-length cDNA of the L1 segment of MRV strain T3D [28]. Viral cDNA fused at their native 5′-termini to the T7 promoter were inserted into pT7-L1T3D by complete replacement of plasmid sequences encoding T3D L1, resulting in ligation of native 3′-termini to the HDV ribozyme sequence. pT7-S3MB, encoding the entire MB S3 gene, has an EcoRV restriction site as a genetic marker created by introducing a single nucleotide change at position 640 in the S3 gene using a KOD-Plus-Mutagenesis kit (Toyobo). To generate the rescue plasmids pT7-S1NB and pT7-S1Mel, encoding strains NB and Mel S1 genes, respectively, the full-length cDNAs of NB S1 and Mel S1 genes were synthesized by gene synthesis services (Eurofins Genomics) based on the nucleotide sequences of NB S1 (GenBank accession number: AF218360) and Mel S1 (GenBank accession number: EF026043), respectively. The artificial synthetic NB S1 and Mel S1 cDNAs were inserted into pT7-L1T3D, thereby replacing the T3D L1 cDNA and generating pT7-S1NB and pT7-S1Mel, respectively. To generate constructs for the rescue of σC mutant viruses, pT7-S1MB was altered using a KOD-Plus-Mutagenesis kit. pT7-S1MB-σC-del contains a deletion of the S1 nucleotide sequence 707–1450 within the σC ORF. To generate pT7-S1MB-σC-ACG, the start codon and five other downstream AUG codons in the σC ORF were disrupted (AUG–ACG) at nucleotide positions 572–574, 581–583, 653–655, 716–718, 1010–1012, and 1121–1123, and the five stop codons were inserted into the σC ORF. The coding sequence of the overlapping p17 ORF was not affected. To generate pT7-S1MB-Head-del, lacking the C-terminal region of σC protein, a stop codon (UCA–UGA) was introduced into the S1 cDNA at the nucleotide position 1160–1162. To generate constructs for the rescue of monoreassortant viruses that contain NB or Mel S1 segments lacking σC protein expression, a KOD-Plus-Mutagenesis kit was used. Rescue plasmids pT7-S1NB-σC-del and pT7-S1Mel-σC-del contain deletions of the S1 nucleotide sequences 700–1466 and 709–1454 within the σC ORF, respectively. pT7-S1NB-σC-ACG and pT7-S1Mel-σC-ACG contain an AUG–ACG modification of the σC translation initiation codon at S1 nucleotide positions 611–613 and 584–586, respectively, and two stop codons were inserted into the σC ORF. To generate pT7-S1MB-σC-ZsY, S1 the nucleotide sequence 1001–1516 within pT7-S1MB was replaced with the ZsYellow ORF. To generate the mammalian expression vector pCAG-MB-σC-FLAG encoding the MB σC protein fused to a copy of FLAG epitope tag at the C-terminus, σC cDNA fused to the FLAG epitope tag was cloned into the EcoRI site of the pCAGGS vector [76]. To generate the mammalian expression vectors p3×FLAG-MB-σC and p3×FLAG-T3D-σ1 encoding the MB σC and MRV T3D σ1 proteins, respectively, fused to three copies of FLAG epitope tag at the N-terminus, MB σC and T3D σ1 cDNAs fused to the FLAG epitope tags were cloned into the EcoRV site and KpnI site, respectively, of the p3×FLAG-CMV-10 expression vector (Sigma). To generate p3×FLAG-Fd-σC-T and p3×FLAG-Fd-σC-BH encoding the N-terminal predicted tail domain (MB S1 nucleotide sequences 572–1006) and the C-terminal predicted head–body domains (MB S1 nucleotide sequences 1007–1567) of σC protein, respectively, partial σC cDNA fragments fused to a foldon sequence (GYIPEAPRDGQAYVRKDGEWVLLSTFL), derived from the T4 phage fibritin, at the N-terminus to stabilize the protein trimer were cloned into the EcoRV and KpnI sites of the p3×FLAG-CMV-10 expression vector [53]. The nucleotide sequences of the plasmids were confirmed by DNA sequencing. The primer sequences used for plasmid construction are available upon request.

### Recovery of recombinant viruses from cloned cDNA

Monolayers of L929 cells (8 × 10^5^ cells) in six-well plates (Corning) were infected with rDIs-T7pol at an MOI of ~3 TCID_50_/cell. At 1 h post infection, cells were cotransfected with plasmids encoding each of the 10 gene segments of strain MB (pT7-L1MB, 0.66 μg; pT7-L2MB, 0.66 μg; pT7-L3MB, 0.66 μg; pT7-M1MB, 0.58 μg; pT7-M2MB, 0.58 μg; pT7-M3MB, 0.58 μg; pT7-S1MB, 0.5 μg; pT7-S2MB, 0.5 μg; pT7-S3MB, 0.5 μg; and pT7-S4MB, 0.5 μg) using 2 μl of TransIT-LT1 transfection reagent (Mirus) per microgram of plasmid DNA. Following 1–2 days of incubation, recombinant virus was isolated from transfected cells by plaque purification using L929 cell monolayers. To establish the vaccinia virus-free reverse genetics system, monolayers of BHK/T7-9 cells (8 × 10^5^ cells) seeded in six-well plates were cotransfected with the 10 MB plasmids. The amount of each plasmid used for transfection was identical to that described for vaccinia virus-based reverse genetics system. To generate viruses containing engineered changes in σC, cells were cotransfected with nine plasmids from strain MB in combination with pT7-S1MB-σC-del, pT7-S1MB-σC-ACG, pT7-S1NB-σC-del, pT7-S1NB-σC-ACG, pT7-S1Mel-σC-del, pT7-S1Mel-σC-ACG, pT7-S1MB-Head-del, or pT7-S1MB-σC-ZsY. The mutations in the S1 segment from recombinant viruses were confirmed by nucleotide sequence analysis using extracted dsRNA genome from the virions.

### Growth kinetics of recombinant viruses

Monolayers of L929 or Vero cells (2 × 10^5^ cells) in 24-well plates (Corning) were infected with viruses at an MOI of 0.1 PFU/cell. After 1 h of incubation, the cells were washed using phosphate-buffered saline (PBS) twice and incubated with maintenance medium. Cultures were harvested at various intervals for virus titration.

### Syncytium formation

Monolayers of Vero cells (8 × 10^5^ cells) in six-well plate were infected with the viruses at an MOI of 0.1 PFU/cell. After 1 h of incubation, the cells were washed using PBS twice and incubated with maintenance medium for 12 h. The cells were fixed with methanol and stained with Giemsa’s Stain Solution (Nacalai Tesque).

### Electrophoretic analysis of viral RNAs

Viral dsRNAs were extracted from virions and mixed with equal volume of 2 × sample buffer (125 mM Tris–HCl pH 6.8, 10% 2-mercaptoethanol, 4% SDS, 10% sucrose). The dsRNAs were separated using a 10% precast polyacrylamide gel (Atto) and visualized by ethidium bromide staining.

### Generation of antiserums against NBV antigens

To generate antiserum against strain MB σC, the σC coding region of the MB S1 gene was cloned downstream of sequences encoding poly**-**histidine (His) tag in the pTrcHisA vector (Life Technologies). The His-σC fusion protein expressed in BL21 cells (Takara) was purified from the soluble fraction using His-Select R Nickel Affinity Gel (Sigma) according to the manufacturer’s instructions. The His-σC fusion protein was mixed with Alhydrogel adjuvant 2% (InvivoGen) according to the manufacturer’s instructions, and ICR mice (CLEA Japan) were immunized and boosted with the protein-adjuvant mixture to generate σC-specific serum. Antiserum was obtained 4 weeks after administration of the last booster. To generate antiserum against strain MB, virions were mixed with Alhydrogel adjuvant 2% according to the manufacturer’s instructions, and mice were immunized and boosted with the virus-adjuvant mixture. Antiserum was obtained 4 weeks after administration of the last booster.

### Immunoblotting

The cells were lysed in buffer consisting of 25 mM Tris–HCl pH7.4, 150 mM NaCl, 1% NP-40, 1% sodium deoxycholate, and 0.1% SDS. After centrifugation, the soluble protein fractions were size fractionated using SDS-polyacrylamide gel electrophoresis and electroblotted onto polyvinylidene difluoride membranes (Millipore). Viral proteins were detected using Chemi-Lumi One Ultra (Nacalai Tesque) following incubation with antiserum-specific for σC or NBV at a dilution of 1:2000 and HRP conjugated anti-mouse IgG secondary antibody (Sigma) at a dilution of 1:2000.

### Immunofluorescence and infectivity assays

Monolayers of cells were seeded onto glass coverslips (As One) and infected with the viruses. After incubation of 12 h for L929 cells or 6 h for A549 cells, cells were fixed with PBS containing 4% paraformaldehyde, washed with PBS, and incubated with NBV-specific antiserum. After three washes with PBS, cells were incubated with CF488 Goat Anti-Mouse IgG second antibody (Biotium) or Alexa Fluor 633 Goat Anti-Mouse IgG second antibody (Invitrogen) at a dilution of 1:1000. Cells were also incubated with 4′,6-diamidino-2-phenylindole (DAPI) to label nuclei and then washed three times using PBS. For infection inhibition assay using σC-specific antiserum, the antiserum was added to virus stock at various concentrations and incubated for 1 h prior to infection. The images were acquired with a FluoView FV1000 laser scanning confocal microscope (Olympus). The number of nuclei in the image was counted using ImageJ software [77]. The infectivity rate of the viruses was expressed as the ratio of the number of infected cells to the total number of cells in the image.

### Expression and purification of the soluble reovirus cell attachment proteins

To express the cell attachment proteins MRV σ1 and NBV σC in mammalian cells, 293T cells were transfected with p3×FLAG-T3D-σ1 or p3×FLAG-MB-σC using 1 mg/ml polyethyleneimine solution (Cosmo Bio). After 48 h of incubation, the cells were collected and lysed in buffer containing of 50 mM Tris–HCl pH 7.4, 150 mM NaCl, and 1% Triton X-100. The recombinant proteins were purified from the soluble fraction using ANTI-FLAG M2 Affinity Gel (Sigma) according to the manufacturer’s instructions. The purified proteins were competitively eluted using 3 × FLAG peptide (Sigma). The proteins were dialyzed using Vivaspin 6 (Sartorius) and used for cell-surface binding assays.

### Cell-surface binding assay

The cells were detached using Cell Dissociation Solution Non-enzymatic (Sigma). In total, 5 × 10^5^ cells were incubated with purified soluble recombinant proteins at 4°C for 1 h. The cells were washed three times using PBS and incubated with monoclonal anti-FLAG-M2 antibody (Sigma) at a dilution of 1:500 at 4°C for 1 h. After three washes with PBS, the cells were incubated with CF488 Goat Anti-Mouse IgG second antibody at a dilution of 1:500 at 4°C for 1 h. The cells were incubated with PBS containing 10 μg/ml propidium iodide solution (Sigma) to stain dead cells. The signal intensity of living cells was quantified using a FACSCalibur (Becton Dickinson). Data were analyzed using FlowJo software.

### Infection of mice

To evaluate the role of σC protein in viral pathogenesis, a mouse model of NBV infection was used (Y. Kanai and T. Kobayashi, manuscript in preparation). Four-week-old male C3H mice were purchased from CLEA, Japan. The mice were intranasally infected with 20 μl (4 × 10^5^ PFU) of purified virus diluted with PBS and their body weight changes and survival were monitored for 14 days. Mice were euthanized when moribund.

### Statistical analysis

Each data point is expressed as the mean of triplicate samples. Error bars indicate the standard deviation. The significance of differences was determined via Student’s *t*-test or ANOVA or log rank test using Prism software (GraphPad Software, Inc.). p values < 0.05 were considered statistically significant.

### Ethics statement

The mouse experiments were conducted following the approval of the Animal Research Committee of Research Institute for Microbial Diseases, Osaka University and the guidelines for the Care and Use of Laboratory Animals of the Ministry of Education, Culture, Sports, Science and Technology, Japan.

## Supporting Information

S1 Fig Recovery of monoreassortant viruses containing the S1 gene lacking σC expression. (A) Expression of σC in purified wild-type virions. L929 cells were transfected with pCAG-MB-σC-FLAG or infected with rsMB. Purified virions were prepared from cell lysates by equilibrium density ultracentrifugation in CsCl gradients. The cell lysates or virions were analyzed by immunoblotting using σC-specific antiserum or antibody specific for actin. The molecular weights of the proteins are shown in kilodaltons (kDa). (B) Schematic presentation of NB and Mel S1 segments of rsMB/NB-S1-σC-ACG, rsMB/NB-S1-σC-del, rsMB/Mel-S1-σC-ACG, and rsMB/Mel-S1-σC-del (pT7-S1NB-σC-ACG, pT7-S1NB-σC-del, pT7-S1Mel-σC-ACG, and pT7-S1Mel-σC-del, respectively). The black arrowheads indicate the disrupted start codon of the NB and Mel σC proteins. The red arrowheads indicate the stop codon mutation sites. (C) Electropherotype of the dsRNA of the viruses. The viral dsRNA was extracted from purified virions, electrophoresed, and visualized by ethidium bromide staining. Classes of gene segments based on their sizes are indicated.
(TIF)

S2 Fig Caspase activity of rsMB and σC-deficient viruses in L929 cells. To assess the involvement of σC in apoptosis induction, the caspase activity of the cells infected with rsMB, rsMB/σC-ACG, or MRV strain rsT3D was determined. A monolayer of L929 cells (1 × 10^5^ cells/well) in 48-well plates was infected with rsMB, rsMB/σC-ACG, or rsT3D at an MOI of 1 PFU/cell. After 1 h of incubation, the cells were washed with PBS once and incubated for various intervals. The caspase activity of the infected cells was measured using the Caspase-Glo 3/7 Assay (Promega) according to the manufacturer’s instructions. The cell viability was measured using the CellTiter-Glo Luminescent Cell Viability Assay (Promega) according to the manufacturer’s instructions. The relative caspase activity of the living cells was determined by calculating the ratio of caspase activity to that of mock infected cells. The results are expressed as the mean for triplicate samples, and the error bars indicate the standard error of the mean. Significant differences in comparison to mock infected cells were identified using two-way ANOVA. NS: not significant; *p < 0.05; **p < 0.005.
(TIF)

S3 Fig The infectivity of rsMB and rsMB/σC-ACG in BHK-21, CHO-K1, DemKT1, and Vero cells. The cells were infected with the viruses at an MOI of 30 PFU/cell and incubated for 12 h. After incubation, the infectivity of the viruses was analyzed by an indirect immunofluorescence assay using NBV-specific polyclonal antiserum.
(TIF)

S4 Fig Involvement of σC for cell attachment. (A) Binding capacity of rsMB in A549 cells. A549 cells were incubated with rsMB, rsMB/σC-ACG, or rsMB pretreated with σC-specific antiserum at an MOI of 1 PFU/cell for 1 h at 4°C. After incubation, the cells were washed with PBS three times and incubated with NBV-specific antiserum at a dilution of 1:500, followed by CF488 Goat Anti-Mouse IgG second antibody at a dilution of 1:500. The cells associated with the virus were quantified using flow cytometry. (B) Expression and purification of 3 × FLAG-T3D-σ1 and 3 × FLAG-MB-σC proteins. 293T cells were transfected with p3×FLAG-T3D-σ1 or p3×FLAG-MB-σC using 1 mg/ml polyethyleneimine solution. After purification of the recombinant proteins from the cell lysate, the proteins were analyzed by immunoblotting using anti-FLAG-M2 antibody. The molecular weights of the proteins are shown in kilodaltons (kDa). (C) Binding capacity of 3 × FLAG-T3D-σ1 or 3 × FLAG-MB-σC to BHK-21, DemKT1, and Vero cells. The cells were incubated with the protein for 1 h, and the number of cells bound by the protein was quantified by flow cytometry.
(TIF)
