# Supplementary figures and images for: Reverse Genetics for Fusogenic Bat-Borne Orthoreovirus Associated with Acute Respiratory Tract Infections in Humans: Role of Outer Capsid Protein σC in Viral Replication and Pathogenesis
Source: PLoS Pathog. 2016 Feb 22;12(2):e1005455. doi: 10.1371/journal.ppat.1005455 (PMC4762779; doi:10.1371/journal.ppat.1005455)

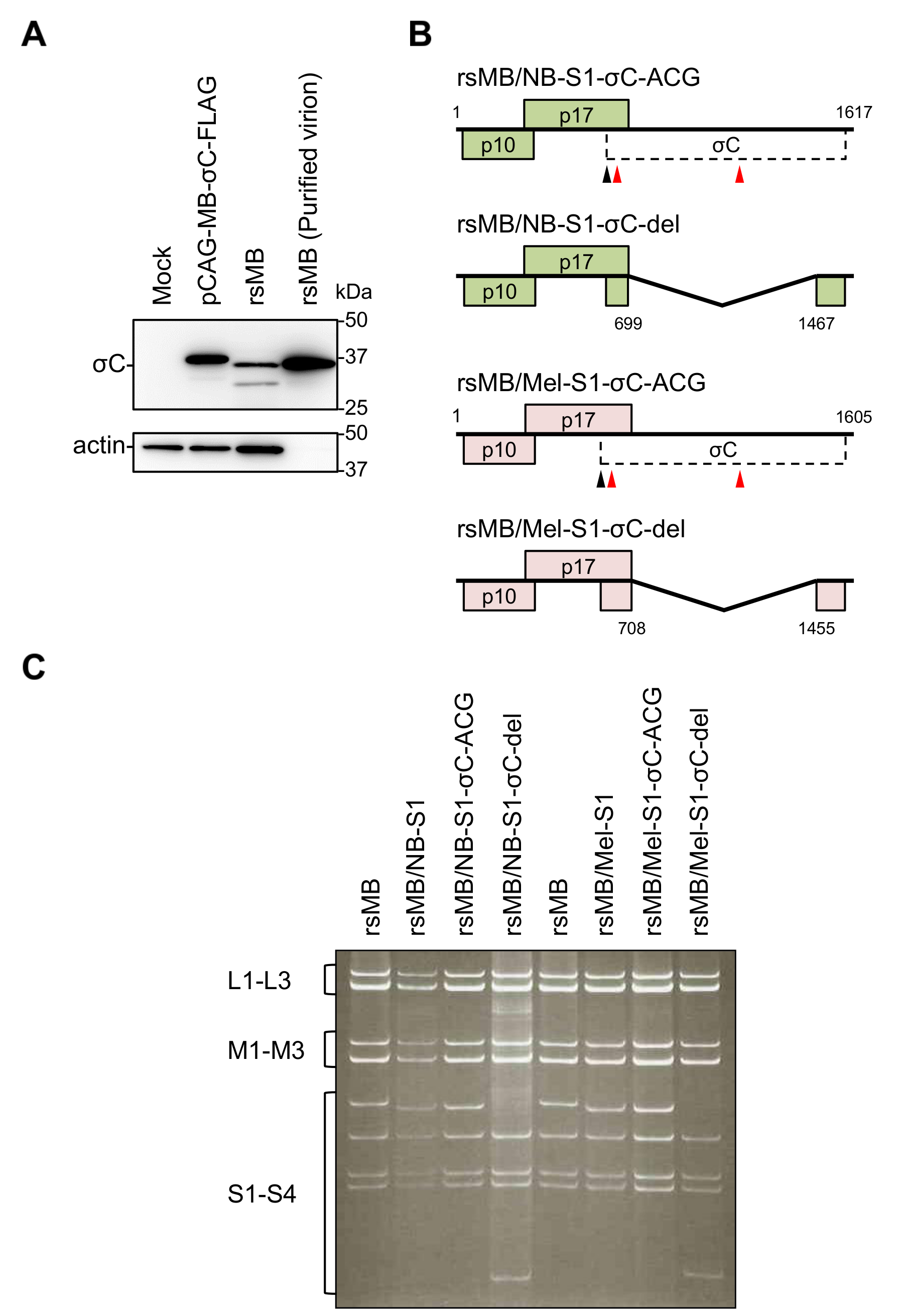

Supplement: S1 Fig — (A) Expression of σC in purified wild-type virions. L929 cells were transfected with pCAG-MB-σC-FLAG or infected with rsMB. Purified virions were prepared from cell lysates by equilibrium density ultracentrifugation in CsCl gradients. The cell lysates or virions were analyzed by immunoblotting using σC-specific antiserum or antibody specific for actin. The molecular weights of the proteins are shown in kilodaltons (kDa). (B) Schematic presentation of NB and Mel S1 segments of rsMB/NB-S1-σC-ACG, rsMB/NB-S1-σC-del, rsMB/Mel-S1-σC-ACG, and rsMB/Mel-S1-σC-del (pT7-S1NB-σC-ACG, pT7-S1NB-σC-del, pT7-S1Mel-σC-ACG, and pT7-S1Mel-σC-del, respectively). The black arrowheads indicate the disrupted start codon of the NB and Mel σC proteins. The red arrowheads indicate the stop codon mutation sites. (C) Electropherotype of the dsRNA of the viruses. The viral dsRNA was extracted from purified virions, electrophoresed, and visualized by ethidium bromide staining. Classes of gene segments based on their sizes are indicated. (TIF) [file ppat.1005455.s001.tif]

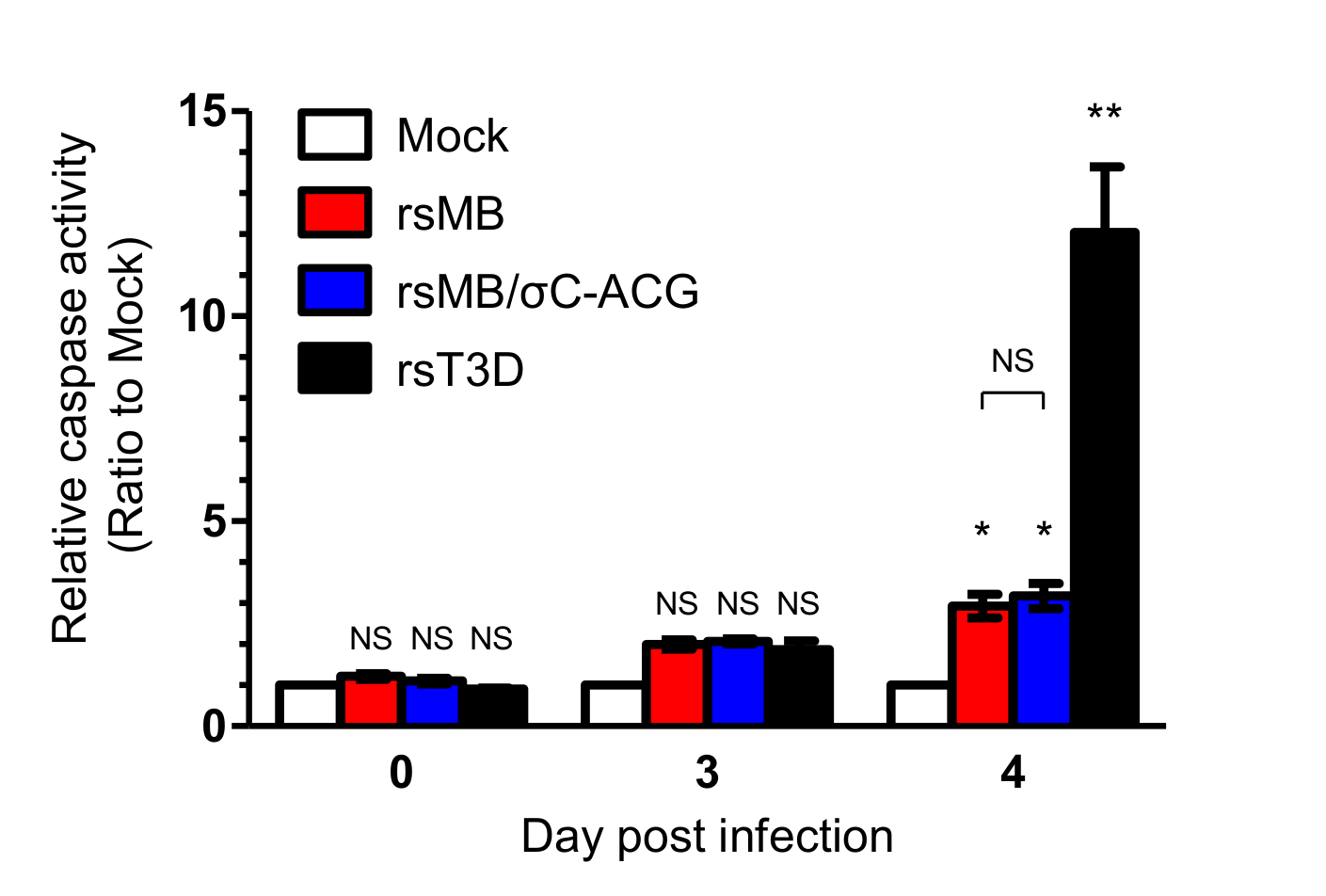

Supplement: S2 Fig — To assess the involvement of σC in apoptosis induction, the caspase activity of the cells infected with rsMB, rsMB/σC-ACG, or MRV strain rsT3D was determined. A monolayer of L929 cells (1 × 10^5^ cells/well) in 48-well plates was infected with rsMB, rsMB/σC-ACG, or rsT3D at an MOI of 1 PFU/cell. After 1 h of incubation, the cells were washed with PBS once and incubated for various intervals. The caspase activity of the infected cells was measured using the Caspase-Glo 3/7 Assay (Promega) according to the manufacturer’s instructions. The cell viability was measured using the CellTiter-Glo Luminescent Cell Viability Assay (Promega) according to the manufacturer’s instructions. The relative caspase activity of the living cells was determined by calculating the ratio of caspase activity to that of mock infected cells. The results are expressed as the mean for triplicate samples, and the error bars indicate the standard error of the mean. Significant differences in comparison to mock infected cells were identified using two-way ANOVA. NS: not significant; *p < 0.05; **p < 0.005. (TIF) [file ppat.1005455.s002.tif]

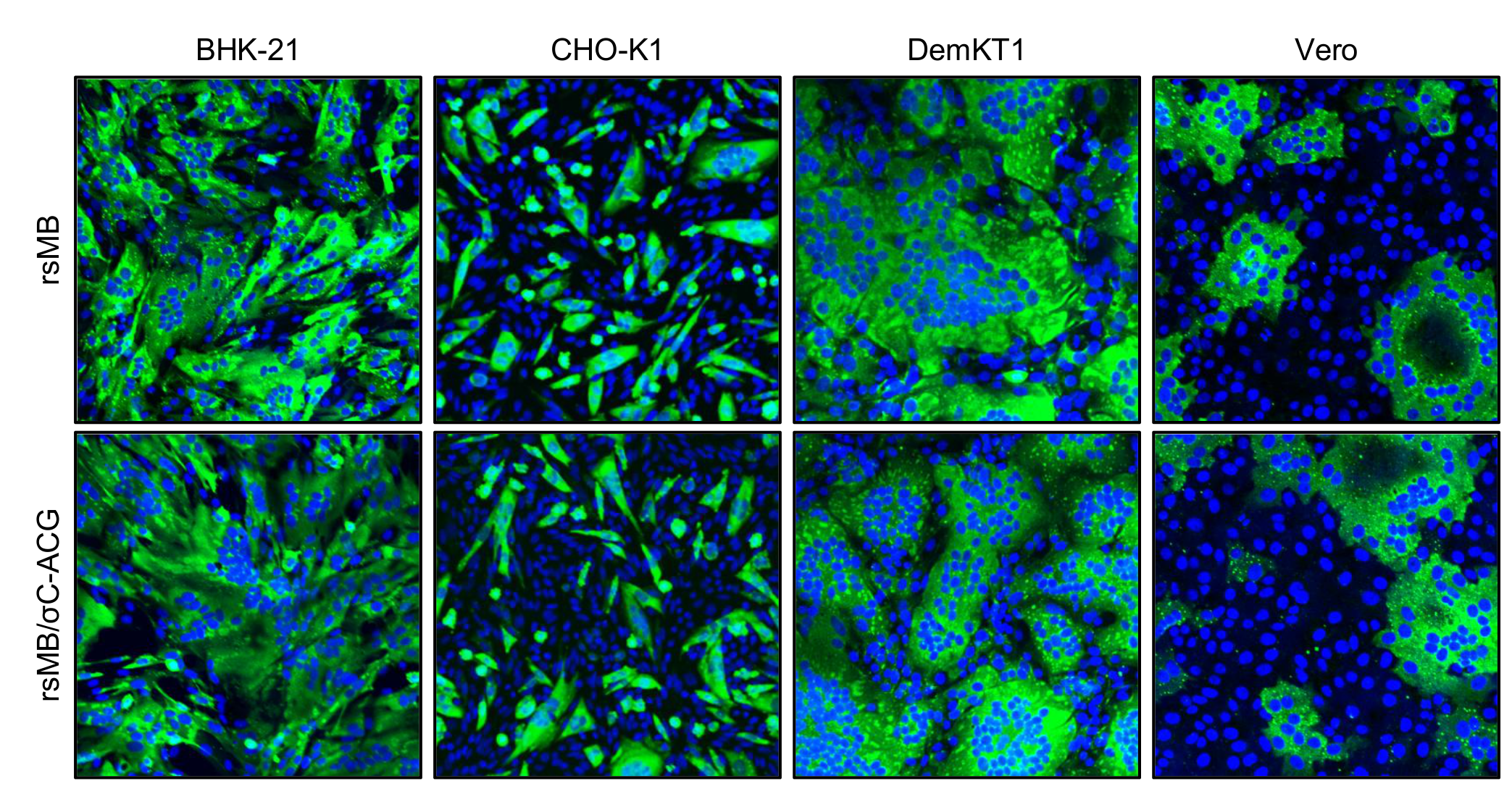

Supplement: S3 Fig — The cells were infected with the viruses at an MOI of 30 PFU/cell and incubated for 12 h. After incubation, the infectivity of the viruses was analyzed by an indirect immunofluorescence assay using NBV-specific polyclonal antiserum. (TIF) [file ppat.1005455.s003.tif]

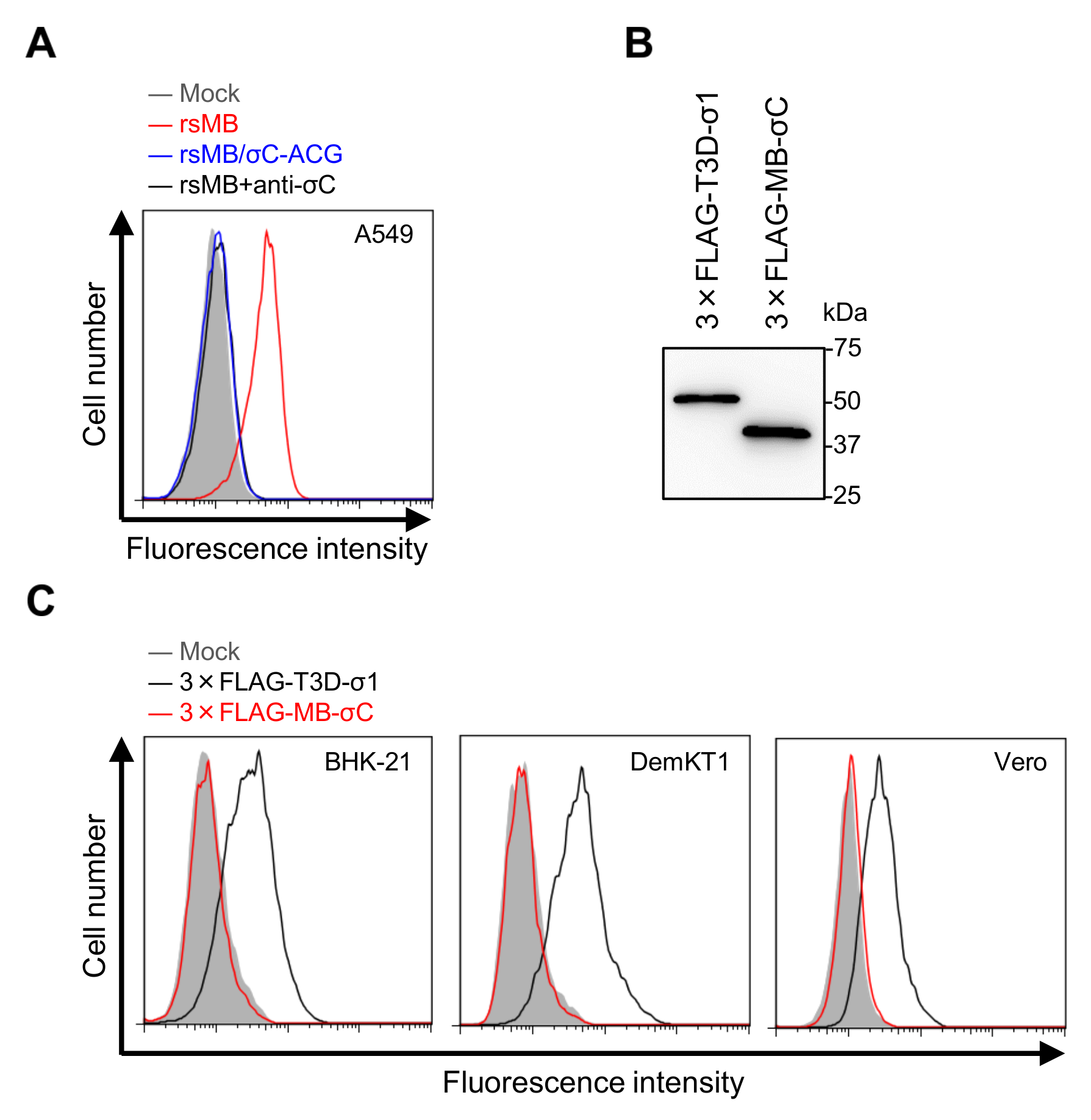

Supplement: S4 Fig — (A) Binding capacity of rsMB in A549 cells. A549 cells were incubated with rsMB, rsMB/σC-ACG, or rsMB pretreated with σC-specific antiserum at an MOI of 1 PFU/cell for 1 h at 4°C. After incubation, the cells were washed with PBS three times and incubated with NBV-specific antiserum at a dilution of 1:500, followed by CF488 Goat Anti-Mouse IgG second antibody at a dilution of 1:500. The cells associated with the virus were quantified using flow cytometry. (B) Expression and purification of 3 × FLAG-T3D-σ1 and 3 × FLAG-MB-σC proteins. 293T cells were transfected with p3×FLAG-T3D-σ1 or p3×FLAG-MB-σC using 1 mg/ml polyethyleneimine solution. After purification of the recombinant proteins from the cell lysate, the proteins were analyzed by immunoblotting using anti-FLAG-M2 antibody. The molecular weights of the proteins are shown in kilodaltons (kDa). (C) Binding capacity of 3 × FLAG-T3D-σ1 or 3 × FLAG-MB-σC to BHK-21, DemKT1, and Vero cells. The cells were incubated with the protein for 1 h, and the number of cells bound by the protein was quantified by flow cytometry. (TIF) [file ppat.1005455.s004.tif]
